# Vulnerability to bipolar disorder is linked to sleep and sleepiness

**DOI:** 10.1038/s41398-019-0632-1

**Published:** 2019-11-11

**Authors:** Tilman Hensch, David Wozniak, Janek Spada, Christian Sander, Christine Ulke, Dirk Alexander Wittekind, Joachim Thiery, Markus Löffler, Philippe Jawinski, Ulrich Hegerl

**Affiliations:** 1 Department of Psychiatry and Psychotherapy, University of Leipzig Medical Center, Leipzig, Germany; 2 LIFE—Leipzig Research Center for Civilization Diseases, Universität Leipzig, Leipzig, Germany; 3 Depression Research Centre, German Depression Foundation, Frankfurt am Main, Germany; 4 Institute of Laboratory Medicine, Clinical Chemistry and Molecular Diagnostics, University of Leipzig Medical Center, Leipzig, Germany; 5 Institute for Medical Informatics, Statistics and Epidemiology (IMISE), University of Leipzig, Leipzig, Germany; 6 Department of Psychology, Humboldt-Universität zu Berlin, Berlin, Germany; 7 Department of Psychiatry, Psychosomatic Medicine and Psychotherapy, Goethe-Universität Frankfurt, Frankfurt am Main, Germany

**Keywords:** Predictive markers, Bipolar disorder, Pathogenesis, Human behaviour

## Abstract

Sleep impairments are a hallmark of acute bipolar disorder (BD) episodes and are present even in the euthymic state. Studying healthy subjects who are vulnerable to BD can improve our understanding of whether sleep impairment is a predisposing factor. Therefore, we investigated whether vulnerability to BD, dimensionally assessed by the hypomanic personality scale (HPS), is associated with sleep disturbances in healthy subjects. We analyzed participants from a population-based cohort who had completed the HPS and had either a 7-day actigraphy recording or a Pittsburgh sleep quality index (PSQI) assessment. In addition, subjects had to be free of confounding diseases or medications. This resulted in 771 subjects for actigraphy and 1766 for PSQI analyses. We found strong evidence that higher HPS scores are associated with greater intraindividual sleep variability, more disturbed sleep and more daytime sleepiness. In addition, factor analyses revealed that core hypomanic features were especially associated with self-reported sleep impairments. Results support the assumption of disturbed sleep as a possibly predisposing factor for BD and suggest sleep improvement as a potential early prevention target.

## Introduction

Sleep disturbances and increased daytime sleepiness occur in bipolar disorder (BD), including in the euthymic state^1,2^. Accumulating evidence suggests that impaired or reduced sleep and increased daytime sleepiness are not only symptoms of BD, but contribute to the disease process itself and to (hypo)manic behavior in particular. Experimental and longitudinal studies of patients, healthy subjects, and animals suggest that sleep deprivation can induce (hypo)mania^3–9^. Accordingly, cognitive behavioral therapy for inter-episode insomnia has resulted in a lower (hypo)manic relapse rate^10^, and stabilization of sleep and sleep–wake rhythms is an element of BD treatments^11,12^. Further, in a recent study^13^ daytime sleepiness predicted (hypo)manic relapse. Daytime sleepiness has been characterized as the behavioral expression of an unstable central nervous system (CNS)-arousal;^14^ and using electroencephalogram (EEG), unstable arousal has been demonstrated for BD, especially during mania^15–18^. The arousal regulation model of affective disorders and attention-deficit/hyperactivity disorder (ADHD)^15,17^ takes into consideration this unstable arousal in BD (and ADHD) and suggests that hypoarousal can partly cause (hypo)manic behavior. In an autoregulatory manner, the hyperactive and sensation-seeking behavior is seen as a compensatory arousal-stabilizing behavior, which can in turn increase sleep deficits, thus initiating a vicious circle contributing to mania^15,17,18^.

If impaired sleep is in fact a predisposing trait for BD, sleep improvement might be a target for early interventions. However, present findings on impaired sleep in euthymic BD leave it unclear to what extent these disturbances are a predisposition, a consequence of the disease process, or due to medication. For example, BD patients are often prescribed numerous medications^19^, many of which impact both arousal and sleep. Studying healthy subjects who are vulnerable to BD can improve our understanding of whether sleep impairment is a predisposing factor. *Genetic* high-risk studies have mostly shown that sleep in healthy offspring of BD patients is indeed impaired^20–22^. However, in addition to the small sample sizes in many of these studies, a further inherent limitation is the exclusive focus on a particular subgroup, namely offspring of relatives who have fallen ill. There is only one longitudinal study, which was not based on genetic high-risk offspring but on a healthy community sample, and which could nonetheless predict subsequent development of BD during a ten year follow up by poor sleep quality at baseline^23^. Moreover, this prediction of BD by poor sleep persisted when excluding subjects with a family history of affective disorders. However, results of this study were based on only 20 subjects who developed a full-blown BD. Missing data and a low probability of incident BD in healthy subjects not genetically at-risk are inherent problems of such longitudinal cohort studies. Thus, more data are needed. The *psychometric* high-risk approach^24^, which assesses BD risk in large populations via questionnaires such as the hypomanic personality scale (HPS)^25^, is one useful method. The HPS allows not only the identification of unusually high scorers (high risk subjects), but also the assessment of the total normal variance. The association of the entire continuum of bipolarity with sleep disturbances can then be examined. Such dimensional concepts of psychopathology have recently attracted much interest in the context of psychiatric genetics and the endophenotype approach^26–28^, and also the Research Domain Criteria Project (RDoC) of the National Institute of Mental Health (NIMH) pointed out the strength of dimensional assessments^29^.

The HPS assesses features of hypomanic episodes as described within the diagnostic systems, but also includes correlated traits such as social dominance. As the scale assesses a temperamental dimension, i.e., a personality trait rather than an acute episode, subjects are instructed to respond how they feel in general. Several studies have demonstrated the scale’s reliability and validity. The HPS is stable over time^25,30^, has been associated with psychiatric risk genes^31,32^, aggregates in families of afflicted patients^33^ and has discriminated bipolar patients from controls^34,35^. Cross-sectionally, HPS high scorers showed higher rates of (hypo)manic and depressed episodes and more psychosocial impairment and substance-use^25,36,37^. Longitudinally, the HPS predicted BD and hypomanic symptoms in addition to related disorders such as substance abuse^38–40^.

Further evidence for the role of hypomanic traits in BD have been presented by other authors such as Hagop S. Akiskal and Richard A. Depue^41^. Akiskal developed an interview (TEMPS-I^42^) and self-rating instrument (TEMPS-A^43,44^) in which the hyperthymic and cyclothymic temperaments measure comparable constructs to the HPS^45,46^. As is the case with the HPS, the TEMPS-A temperaments show long-term stability^47^, aggregate in families^27,48^, have been associated with psychiatric risk genes^49^ and have been used to discriminate and predict bipolar symptomatology^48,50^. One difference between the TEMPS-A and the HPS is that the TEMPS-A assesses a hyperthymic temperament separately from a cyclothymic temperament, whereas in the HPS both aspects are summed together in one total score. However, it has recently been suggested that the HPS should be separated into different subscales which may have divergent psychopathological correlates^51–53^. In addition to the research on affective temperament scales such as HPS and TEMPS-A, longitudinal studies have contributed further evidence that subthreshold hypomanic symptoms often precede BD^39,54–58^.

To date, only two small studies using student samples have assessed the association between sleep and HPS. In HPS high-scorers, greater intraindividual variability in sleep duration was observed via sleep diaries^59^ and actigraphy^60^. The latter study^60^ also showed shorter sleep duration and greater intraindividual variability in sleep efficiency in HPS high-scorers. As is usually the case, these two studies utilized the HPS total sum-score. However, in two recent studies^51,53^ HPS subscales were derived from factor analyses which differed in their associations with psychopathologically relevant traits^51,53,61^.

### Objectives

The current study analyses the associations of objective and subjective sleep parameters and daytime sleepiness with a risk factor for BD, the HPS. In a large cohort of healthy subjects, we expect associations between the HPS and shorter and more disturbed sleep, increased daytime sleepiness and more night-to-night sleep-variability. In contrast to most prior research, we not only associate sleep with the HPS total score, but also with HPS subscales. To this end, we conducted the first factor analysis on the German translation^62^ of the HPS. We hypothesize that the associations of the HPS with sleep variables will differ with respect to the HPS subscales.

## Materials and methods

### Participants

The study sample was drawn from the LIFE-Adult study^63^, a population-based cohort comprised of 10,000 inhabitants of the city of Leipzig, Germany. Of the total sample, 3031 participants aged 60–82 years completed the HPS. Subjects had to be free of diseases or medications which could strongly impact sleep–wake behavior. Thus, participants with a history of stroke, multiple sclerosis, Parkinson’s disease, epilepsy, skull fracture, cerebral tumor, or meningitis were excluded, leaving 2788 subjects. Further excluded were individuals reporting current use of CNS-affecting drugs (leaving 2373 subjects). Based on data from structured clinical interviews for DSM-IV Axis I disorders, we selected subjects without a lifetime history of substance dependence, psychotic or BDs, and who were free of current affective or anxiety disorders (leaving 2087 subjects). In addition, participants were required to have available data from either the Pittsburgh sleep quality index (PSQI) assessment or an actigraphy recording for at least five nights. This resulted in a final sample of 771 subjects for actigraphy association analyses (372 female, M_age_ = 70.3 y), and 1766 subjects for PSQI association analyses (835 female, M_age_ = 69.6 y). Factor analyses of the HPS were conducted using all 2861 subjects with complete HPS responses (1371 female; age range: 60–82 years; M_age_ = 70.0). The study was approved by Leipzig University’s Ethics Committee (263-2009-14122009) and subjects gave written informed consent.

### Objective and subjective sleep assessment

To obtain objective measurements of sleep, subjects wore the SenseWear Pro 3 Armband actigraph (BodyMedia; Pittsburgh, Pennsylvania) for an average of 6.9 days (range: 5–7 days). Analyses of night-sleep parameters were carried out as described in detail elsewhere^64–66^. Subjective ratings of sleep and sleep quality were obtained using the German version of the PSQI^67^ a self-rating instrument to assess sleep quality during the past 4 weeks.

### Hypomanic personality scale (HPS)

The HPS, a self-rating scale used to assess BD risk^25^, was developed in undergraduates, which is reflected in some of the 48 items. As the current study administered the HPS to elderly subjects, four items from the German translation^62^ were deleted for reasons of compliance (see Supplementary Methods).

### Statistical analysis

The factorial structure of the HPS was analyzed using the function irt.fa of R package psych (version 1.7.8) as described in Supplementary Methods. Remaining statistical analyses were performed using SPSS 22 (IBM; Armonk, New York). Associations between the sleep variables and the HPS were conducted for both the HPS total sum-score (*HPS total*) and factor scores for each subscale. We conducted partial Spearman correlations adjusting for sex and age. In order to confirm the results, we additionally compared the top and bottom decile HPS groups using Kruskal–Wallis tests.

## Results

### Factorial structure of the HPS

In the Supplementum, factor analysis results are described in detail and compared to the available factor solutions reported in two younger non-German samples^51,68^. Results revealed three factors (see Supplementary Figs. S1–S3), which were well in line with prior studies (see Supplementary Table S1). We labeled the first factor *hypomanic core*, as it was comprised of items with clearly hypomanic content. Items loading high on the second factor (*social vitality*) describe high self-confidence, social dominance, and leadership. The third factor appears to reflect a characteristic that has previously been recognized as “ordinariness”, as it describes balanced and controlled people who consider themselves as average persons^68^. Thus, we retained the term *ordinariness*.

### Correlation analyses

**Fig. 1:**
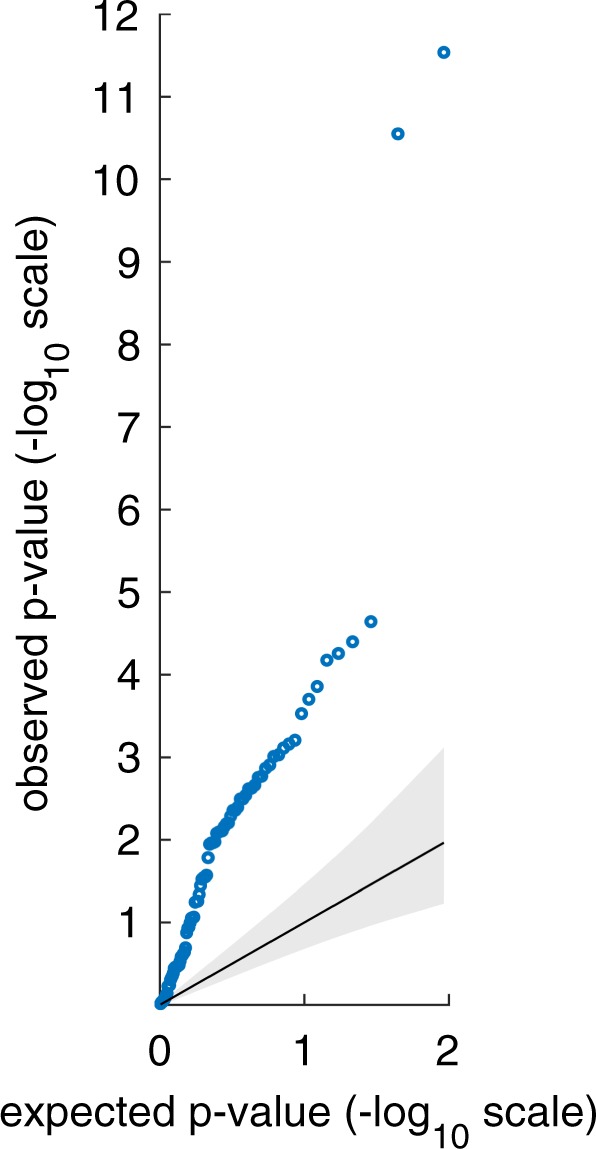
Permutation-based quantile–quantile plot showing that the observed *p* values (blue circles) considerably differ from a random distribution under the null hypothesis (solid diagonal line). For the set of 84 observed *p* values (21 sleep variables × 4 personality scores), one million sets of 84 expected *p* values were derived after data permutation. During data permutation, original correlations within the domain of hypomanic personality variables and the domain of sleep variables were preserved while original correlations between the two domains were removed through random shuffling. Each set of *p* values was sorted in descending order. The solid diagonal line represents the mean expected *p* values at rank 1–84 plotted against themselves. The upper and lower bound of the gray area represent the 5th and 95th percentile of expected *p* values plotted against the mean expected *p* values. The blue circles represent the observed *p* values plotted against the mean expected *p* values

Table 1 shows the Spearman correlations between the sleep parameters and *HPS total* as well as the subscales’ factor scores *hypomanic core*, *social vitality*, and *ordinariness*. In total, 46 out of 84 correlations reached the level of significance, with 40 of them remaining significant after multiple-test correction (see Supplementary Table S2). Accordingly, the quantile–quantile plot suggests that the distribution of observed *p* values considerably differs from a *p* value distribution under the null hypothesis (Fig. 1).

**Table 1:** Partial Spearman correlations between hypomanic personality and sleep–wake variables

	HPS total sum-score		HPS subscale hypomanic core		HPS subscale social vitality		HPS subscale ordinariness	
	rho	*p*	rho	*p*	rho	*p*	rho	*p*
*Actigraphy (n* *=* *771)*								
Means								
Sleep-onset latency	0.038	0.294	0.042	0.240	0.054	0.134	−0.035	0.329
Sleep-onset time	−0.006	0.864	−0.035	0.329	0.025	0.497	0.061	0.089
Sleep-offset time	−0.013	0.721	−0.041	0.252	0.007	0.851	0.056	0.118
Sleep duration	−0.079	0.029*	−0.072	0.046*	−0.059	0.101	0.034	0.350
NWAK	0.078	0.030*	0.069	0.056	0.069	0.056	−0.011	0.757
WASO	0.103	0.004**	0.091	0.011*	0.080	0.027*	−0.046	0.203
Sleep efficiency	−0.106	0.003**	−0.101	0.005**	−0.086	0.017*	0.033	0.361
Night-to-night variability								
Sleep-onset latency	0.019	0.594	−0.005	0.893	0.076	0.036*	−0.026	0.470
Sleep-onset time	0.122	7E−4**	0.102	0.004**	0.113	0.002**	0.009	0.813
Sleep-offset time	0.110	0.002**	0.119	9E−4**	0.069	0.057	−0.062	0.087
Sleep duration	0.098	0.006**	0.092	0.011*	0.097	0.007**	−0.003	0.931
NWAK	0.145	6E−5**	0.109	0.002**	0.152	2E−5**	−0.092	0.011**
WASO	0.115	0.001**	0.116	0.001**	0.095	0.008**	−0.109	0.002**
Sleep efficiency	0.106	0.003**	0.095	0.008**	0.103	0.004**	−0.092	0.011**
*PSQI (n* *=* *1766)*								
Sleep-onset latency^a^	0.013	0.576	0.078	0.001**	−0.080	8E−4**	−0.098	4E−5**
Bedtime^a,b^	0.029	0.230	0.018	0.444	0.041	0.087	0.001	0.968
Get-up time^a^	−0.022	0.348	−0.023	0.344	−0.020	0.413	−0.013	0.581
Sleep duration^a^	−0.038	0.114	−0.095	7E−5**	0.027	0.261	0.081	6E−4**
Sleep efficiency^c^	0.002	0.925	−0.065	0.006**	0.071	0.003**	0.091	1E−4**
Daytime sleepiness^a^	0.075	0.002**	0.088	2E−4**	0.052	0.028*	−0.086	3E−4**
PSQI score^d^	0.063	0.008**	0.158	3E−11**	−0.063	0.008**	−0.165	3E−12**

Night-to-night variability is operationalized by intraindividual standard deviation (ISD) across a single subject’s multiple nights.

Note that *hypomanic core, social vitality and ordinariness* here refer to factor scores derived from factor analyses as described in the Methods of the Supplementum. Results were additionally confirmed by analyses with traditional sum scores (see Supplementary Table S4).

Effects of sex and age were partialled out. *NWAK* number of awakenings, *WASO* wake after sleep-onset time

*p < 0.05

**p < 0.01

^a^Based on the respective Pittsburgh sleep quality index (PSQI) item

^b^Time subject goes to bed

^c^Quotient of sleep duration and time in bed with the latter calculated from bedtime and get-up time

^d^PSQI total score calculated according the manual from all PSQI components; higher PSQI scores mean worse sleep quality

### Objective sleep data

*HPS total* was significantly associated with shorter sleep duration, a greater number of awakenings, more time awake after sleep-onset (WASO) and lower sleep efficiency. At the subscale level, these sleep variables were also significantly associated in the same direction with *hypomanic core*, with the exception of number of awakenings, which narrowly missed the significance level. Associations of *social vitality* were similar, but only WASO and sleep efficiency reached significance. *Ordinariness*, in contrast, was not correlated with any of these actigraphic sleep variables.

*HPS total* was even more strongly associated with the night-to-night *variability* of the sleep parameters than with the means. *HPS total*, *hypomanic core* and *social vitality* were all associated with greater intraindividual variability in sleep-onset time, sleep duration, number of awakenings, WASO and sleep efficiency. In contrast, *Ordinariness* was associated with lower intraindividual variability in the number of awakenings, WASO, and sleep efficiency.

### Subjective sleep data (PSQI)

*HPS total* correlated significantly with more daytime sleepiness and lower sleep quality. Even stronger associations with PSQI-variables were found for *hypomanic core*, which was associated with a longer latency of sleep-onset, shorter sleep duration, lower sleep efficiency, more daytime sleepiness, and lower sleep quality. The obtained correlation coefficients for *hypomanic core* were significantly higher than those of the *HPS total* (Supplementary Table S3), with the exception of daytime sleepiness which did not significantly differ in its positive associations with *HPS total* and *hypomanic core*.

In contrast to *HPS total* and *hypomanic core*, *social vitality*, and *ordinariness* were associated with better subjective sleep and accordingly, both scales’ correlation coefficients differed significantly from *HPS total* and *hypomanic core* (Table S3). *Social vitality* and *ordinariness* were associated with shorter sleep-onset latency, higher sleep efficiency, and better sleep quality. In addition, *ordinariness* also correlated with longer sleep duration and lower daytime sleepiness, while *social vitality* resembled *hypomanic core* in terms of its association with more daytime sleepiness.

### Additional analyses for confirmation of correlation results

**Fig. 2:**
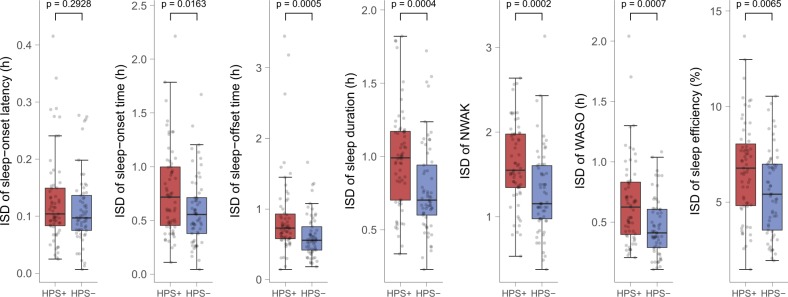
Boxplots of intraindividual night-to-night variability of actigraphic sleep variables stratified by HPS extreme groups. Intraindividual night-to-night variability is operationalized by intraindividual standard deviation (ISD) across a single subject’s multiple nights. Boxplots are stratified by top and bottom decile hypomanic personality scale (HPS) groups (HPS*+*, *N* = 63 vs. HPS−, *N* = 61). Boxes represent the interquartile range of each distribution (data between the lower and upper quartile), with the horizontal line corresponding to the median. Whiskers extend to the furthest observation within 1.5 times the interquartile range from the lower and upper quartile. Dots represent single data points, jittered horizontally to avoid overplotting

Further analyses were carried out to confirm that results were not dependent on the data-analytic method. First, all correlation analyses were repeated with unweighted sum-scores for each HPS subscale instead of factor scores, which resulted in comparable, albeit, as expected, somewhat weaker associations (Supplementary Table S4). Second, analyses were repeated using HPS decile extreme groups. Results resembled those of the correlation analyses (Supplementary Table S5), including the finding that the HPS groups differed more strongly concerning the intraindividual night-to-night variability of the sleep parameters than concerning the means. Figure 2 shows boxplots of the intraindividual sleep variability of actigraphic variables stratified by HPS extreme groups (see Supplementary Fig. S4 for boxplots of all sleep variables). With exception of sleep-onset latency, intraindividual variability for all variables was significantly higher in the top HPS decile group compared with the bottom decile group.

## Discussion

The current study analyzed whether increased vulnerability to BD, as assessed by the HPS, is linked to more disturbed sleep in healthy subjects. Correlation analyses were conducted between objective as well as subjective sleep parameters and HPS subscales and total scale. Extreme group comparisons were also carried out to confirm results.

The correlation analyses as well as the extreme group comparisons revealed that a higher *HPS total* score is associated with worse sleep, greater night-to-night sleep variability and more daytime sleepiness. Thus, results confirm findings of impaired sleep in *genetic* high risk studies^21^.

### Variability matters and HPS subscales differ in their sleep associations

A noteworthy finding is that the HPS was more strongly associated with the night-to-night variability than with the mean sleep variables. In line with this, irregularity in sleep/wake behavior has been reported for euthymic and manic patients^2,69^ and subjects with genetically or psychometrically operationalized heightened BD-risk^2,21,59,60,70,71^. In addition, another study found that greater sleep variability during euthymic state was associated with increased mania and depression severity over 12 months^5^. Thus, our results are in accordance with the role of social/circadian rhythm dysregulation in BD^59,72^.

Another finding is that the HPS subscales differed in their associations with perceived sleep (PSQI). First, the correlations of *hypomanic core* with sleep were significantly higher than the correlations of *HPS total* with sleep. Second, and in contrast to *HPS total* and *hypomanic core*, *ordinariness*, and *social vitality* showed associations in the opposite direction (i.e., they were associated with better perceived sleep). For *ordinariness* this was expected given the content of the scale. However, the association of *social vitality* with better *perceived* sleep is noteworthy, because the subscale is not only positively correlated with *hypomanic core* but also with *objective* sleep impairment. Such diametric associations of the positively correlated HPS facets are in line with the two available studies^51,53^ in which factor-analytic derived HPS subscales showed opposing associations with psychopathologically relevant traits. It might be that subjects scoring high on *hypomanic core* suffer more from impaired sleep, as the first factor (corresponding to *hypomanic core* in our analyses) has been found to be *positively* related to neuroticism^51^, whereas subjects scoring high on *social vitality* might not feel as impaired, given a *negative* association of *social vitality* with neuroticism^51^. Such reversed associations with HPS subscales could cancel each other out in predicting sleep when aggregated in the HPS total sum-score (as we demonstrated in a post-hoc linear regression analysis in Supplementary Results). Thus, the current study strongly supports the suggestion, recently made by several authors, of utilizing HPS subscales^51–53,61^.

In line with the findings of impaired objective sleep in both, *hypomanic core* and *social vitality*, both subscales were associated with increased daytime sleepiness. However, this increased sleepiness seems only to help subjects scoring high on *social vitality* (presumably low in neuroticism) to quickly fall asleep, as was seen in the negative correlation of *social vitality* with sleep-onset latency, whereas *hypomanic core* was associated with longer sleep-onset latency. Thus, *hypomanic core* more strongly resembles long sleep-onset latencies in (euthymic) BD^1,2^ and ADHD^73,74^, which may, despite high sleepiness, occur due to being too hyper and reluctant to fall asleep, rumination, or circadian alterations.

### Sleep duration and daytime sleepiness

The finding that *HPS total* and *hypomanic core* were associated with shorter sleep duration is in line with studies on naturally short sleepers who showed more hypomanic, extraverted and impulsive traits^75,76^. The finding is also consistent with one small study on young HPS high-scorers and a study on children at genetic risk for BD. In both studies, shorter sleep duration was also reported^60,77^. These findings of shorter sleep contrast with the meta-analyses reporting longer sleep in euthymic BD compared to normal controls^1,2^. This might imply that long sleep duration is not a preexisting factor but occurs later as *part* of the disease. In addition, longer sleep could be merely a *consequence* of the disease, as patients might spend more time in bed^13^ as a consequence of sedating medications, unemployment or the instruction to get adequate sleep in order to avoid mania recurrence. Finally, the meta-analyses’ findings of longer sleep during euthymia when measured with actigraphy should be considered with caution, as sleep diaries did not show longer sleep but only longer time-in-bed^2^. It is known that actigraphs based on accelerometers tend to overestimate sleep duration in cases of insomnia symptomatology^78^. This might have contributed to the meta-analyses’ findings of longer sleep (as assessed by actigraphy) in euthymic BD compared to healthy controls. One can speculate that in the current study, because the applied SenseWear Armband uses not only an accelerometer, but also temperature and galvanic skin response sensors to estimate sleep, any potential overestimation of sleep duration might have been less pronounced.

The current study found that greater daytime sleepiness associates with HPS, which is in line with findings of higher sleepiness scores in euthymic BD patients^2^. It has been hypothesized that this sleepiness in BD reflects a *trait-like* arousal instability in some BD patients^15,16^, which would explain the impaired sustained attention in the euthymic state and unaffected relatives^79^. In addition, it has been speculated that the questionable efficacy and mania-inducing properties of antidepressants in BD might partly be attributable to their arousal-reducing properties, which could be problematic if habitual arousal is already low^15,80,81^. One might argue that increased sleepiness should result in longer sleep duration. However, it has been shown that in BD, longer sleep time and daytime sleepiness are independent presentations of hypersomnia^13,82^. Thus, the current findings agree with the assumption that hypoarousal plays a clinically relevant role in BD.

The association of HPS with shorter sleep and greater daytime sleepiness is in line with the arousal regulation model of affective disorders and ADHD, which posits that arousal instability due to short sleep or other reasons can contribute to (hypo)mania and ADHD^15,17,83^. As EEG is an excellent tool to assess arousal^84^, we have developed and validated the publically available Vigilance-Algorithm-Leipzig (VIGALL)^14,85–89^, which allows the assessment of brain arousal in resting-EEG recordings. Utilizing VIGALL, we demonstrated lower arousal during (hypo)mania^18^, and in genetic studies we demonstrated for the first time a link between arousal and ion channels^90,91^, which are of high relevance for BD^92^. ADHD shows symptom overlap with mania and high comorbidity with BD^16,83^. Correspondingly, several studies have also shown both impaired sleep and hypoarousal in ADHD, which may partly explain attention deficits, response to stimulants and compensatory hyperactive behavior^15,83,93^. As in mania, all factors which destabilize arousal such as sleep deficits worsen ADHD symptomatology, whereas sleep improvement is of therapeutic value^15,17,83^.

### Prediction of HPS by sleep and covariates

For several reasons, our analyses focused only on sleep variables. Thus, we did not include variables on the diverse *causes* of disturbed sleep such as sleep apnea. One practical reason for this was that the LIFE cohort comprises only limited data on the causes of disturbed sleep. In fact, only two PSQI items provided information concerning sleep apnea and periodic limb movement disorder (with these two items not aggregated into the PSQI total score). Thus, systematically accounting for sleep disorders can only be done on a relatively weak basis. Most of all, we focused on sleep variables because disturbed sleep has been suggested as the *final common pathway* for a variety of triggers for manic episodes^9^. Sleep disturbances for a variety of reasons (e.g., life events, traveling, leisure activities, circadian dysregulation, drugs, sleep apnea, or periodic limb movement disorder) have been suggested to contribute to (hypo)mania^12^. Finally, we focused our analyses on sleep variables because our goal was to facilitate comparisons of our results for each given sleep variable, such as the WASO, with those from the available literature, particularly the meta-analyses of euthymic BD^1,2^.

Nonetheless, one might ask whether new insights would arise when all variables, including socioeconomic status, age, sex, and body mass index are included simultaneously in multivariable analyses. To investigate the relative importance of objective and subjective sleep variables while accounting for other covariates in predicting HPS scores, we carried out a series of regression analyses. In order to avoid overfitting and inflated *R*^2^ estimates, we pursued a tenfold cross-validation approach which involves evaluating the performance of a prediction model by applying the model to new data not used in training it. Results, which are outlined in the Supplemental in more detail, showed clear incremental predictive value of the sleep variables over the covariates in predicting *HPS total*, *hypomanic core*, and *ordinariness* (Supplementary Table S6). The incremental value of the sleep variables was most pronounced for *hypomanic core*, which is in line with *hypomanic core* being most strongly correlated with sleep in the correlation analyses. In contrast, there was no incremental predictive value of sleep variables on HPS *social vitality*. This finding could be due to the fact that a large proportion of variance in HPS *social vitality* was already explained by the covariate “socioeconomic status” (see Supplementary Fig. S5), which might be considered as further evidence that *social vitality* may be the healthier facet of the HPS^51^

### Strengths and limitations

The HPS has mostly been utilized in samples of adolescents and young adults. To increase the appropriateness of the HPS in our elderly subjects, we deleted four items, thus limiting the comparability of our findings with other studies. However, the factorial structure of the shortened HPS in our sample of German elderly adults largely overlapped with those derived from the total HPS scale in younger samples^51,53,68^, suggesting that the utilized version reveals comparable construct estimates even across different national and age cohorts. Nonetheless, our HPS subscales and their differential associations with sleep variables need replication in other, particularly younger samples. Concerning our subscales’ differential association with BD, we are currently lacking data, but would hypothesize *hypomanic core* to be most strongly associated with BD. There is a large overlap of items from our *hypomanic core* subscale with the items showing the largest correlations with BD diagnoses in a study by Miller et al.^34^. All items from an HPS short version, comprising the six items with the highest BD-association^34^, are part of our *hypomanic core* subscale.

The current study demonstrated that the HPS is also applicable in subjects older than 60 years, which is not trivial given the strong decline of HPS scores with increasing age. For example, in our PSQI sample the mean of the *HPS total* score was M = 9.10 (SD = 5.37; mean extrapolated to a scale length of 48 items). The means and variances of younger samples are substantially higher. In the undergraduate sample by Eckblad and Chapman^25^, the mean was M = 21.74 (SD = 8.16). Similarly, in a German sample^62^ with an age range of 17–30 years, the mean was M = 18 (SD = 8.91). Thus, the current results are compelling in light of the low HPS variance in our sample, which may have made it more difficult to detect associations.

From another perspective, the older age of our sample may be both a limitation and a strength. The aim of our study was to associate the vulnerability factor HPS with sleep, and we were not interested in associations due to effects of current or former affective episodes or treatments. Therefore, we excluded subjects with acute affective episodes, psychotropic drugs, or a life time diagnosis of BD. In addition, our subjects are, due to their age, relatively unlikely to develop BD in the future. This means that, in contrast to studies in younger samples, we can also be quite sure that prodromes of emerging episodes have not biased our results. However, the age and exclusion criteria of the current sample imply that we may have analyzed a sample with superior health, not comparable to the younger samples in which the HPS has mostly been used. Notwithstanding, from a dimensional perspective on psychopathology, it is still reasonable to associate the entire “remaining” vulnerability factor variance with sleep. In addition, such a sample with superior health will still include subjects at high risk for BD. These subjects remained healthy, likely because of a lack of triggering factors or life-events or because of additional protective traits. It is remarkable that in this sample of likely superior health, the HPS is still associated with more impaired sleep and greater sleepiness.

The present study was well-powered and revealed strong evidence for a link between hypomanic temperament and sleep alterations. Nevertheless, observed effect sizes, albeit substantial in extreme group comparisons, were small in correlation analyses across all subjects. One reason for this might be that elderly subjects show low variances in HPS and wake–sleep behavior. Further, during the 7-day actigraphy assessment, sleep was undoubtedly influenced by numerous factors beyond the subject’s predisposition (e.g., common cold, sleep of the partner, and reactivity to the actigraph). Given these sources of error variance, the highly consistent results are very compelling.

## Conclusions

The associations of the HPS with worse sleep were consistent, irrespective of the assessment modality and data analysis method. Sleep inter-night variability showed the most pronounced associations with HPS, thus further qualifying as a characteristic variable in BD^21,70,94,95^. As a further practical implication, this study supports the suggestion recently made by other authors^51–53^ that using HPS subscales will increase the diagnostic power.

The association of sleep alterations with HPS supports assumptions derived from prior studies that sleep disturbances may be a predisposing factor for BD. Analogous to prior studies in which sleep difficulties predicted mood episodes in *genetic* high risk off-spring^70,77,96^, one can hypothesize that sleep might predict conversion to BD in *psychometric* high-risk groups as assessed by the HPS. Early interventions to prevent psychiatric disorders or change their course are a cutting-edge topic in psychiatry^57^. Thus, improving sleep in HPS high-scorers may be a valuable early prevention approach, with sleep symptoms being easy to assess, modifiable, and largely free of social stigmatization.

## Supplementary information

Supplemental Material
