# Supplementary material for: Vulnerability to bipolar disorder is linked to sleep and sleepiness
Source: Transl Psychiatry. 2019 Nov 11;9:294. doi: 10.1038/s41398-019-0632-1 (PMC6848097; doi:10.1038/s41398-019-0632-1)
Supplement: Supplementary file 1 — Supplemental Material [file 41398_2019_632_MOESM1_ESM.docx]

**Supplementary Material**

**Supplementary Methods**

**Participants**

**Hypomanic Personality Scale (HPS)**

**HPS factor analysis**

**Supplementary Results**

**Factorial structure of the HPS**

Supplementary Fig. S1 Scree plot showing the eigenvalues of the principal factors derived from the tetrachoric correlation matrix of HPS items

Supplementary Fig. S2 ICLUST output based on the tetrachoric correlation matrix of HPS items

Supplementary Fig. S3 Tetrachoric correlation matrix of the HPS items

Supplementary Table S1 Loadings and IRT parameters of the HPS three-factor model

**Correlation analyses**

Supplementary Table S2 Partial Spearman correlations between HPS and sleep-wake variables. FDR-corrected and nominal p-values are given for comparison

Supplementary Table S3 Comparison of Spearman correlations

**Secondary analyses for confirmation of results**

Supplementary Table S4 Partial Spearman correlations between HPS (unweighted sum scores) and sleep-wake variables

Supplementary Table S5 HPS total sum score and sleep-wake variables – extreme group comparisons

Supplementary Fig. S4 Boxplots of actigraphy and PSQI variables stratified by HPS extreme groups

**Exploratory regression analysis with HPS subscales**

**Prediction of HPS by sleep and covariates**

Supplementary Table S6 Pearson correlations between predicted and measured HPS scores

Supplementary Fig. S5 Bar plot showing the relative importance of each variable to the prediction model

**Supplementary References**

**Supplementary Methods**

**Hypomanic Personality Scale (HPS)**

We administered the German translation^1^ of the Hypomanic Personality Scale (HPS)^2^. The HPS is a self-rating instrument and is comprised of 48 dichotomous items. As the current study utilized the HPS in elderly subjects aged above 60 years for the first time, the following four inadequate items were deleted for reasons of tolerance and compliance: “I am frequently so ‘hyper’ that my friends kiddingly ask me what drug I’m taking”, “I expect that someday I will succeed in several different professions”, “There are so many fields I could succeed in that it seems a shame to have to pick”, “A hundred years after I’m dead, my achievements will probably have been forgotten”.

Our HPS total sum score, with a possible maximum of 44, ranged from 0 to 37 (mean: 8.34, SD: 5.37) in the PSQI sample and from 0 to 37 (mean: 8.55, SD: 5.59) in the actigraphy sample. Extrapolated to a scale comprised of 48 items, the means were M = 9.10 and M = 9.33, respectively.

**HPS factor analysis**

Factor analyses were conducted using all 2,861 subjects with complete HPS responses (1371 female; age range: 60-82 years; mean age: 70.0).

Given the dichotomous nature of the HPS items, a tetrachoric correlation matrix was performed first. Subsequently, minimum residual factoring was carried out followed by promax rotation. The number of factors was determined by scree plot inspection, the Very Simple Structure (VSS) criterion for complexity one and two, and Velicer’s Minimum Average Partial (MAP) criterion. We also assessed the congruency of the factor solution with results of a hierarchical cluster analysis that was carried out with the ICLUST function of the R package psych^3^. Factor scores were calculated using the function score.irt.2, which uses the two parameter Item Response Theory (IRT) equivalent of loadings and difficulties. Factor score calculations were based on those items showing discrimination parameters higher than or equal to the cut-off value 0.300.

**Supplementary Results**

**Factorial structure of the HPS**

The scree test suggested a three-factor solution (Fig. S1). The other criteria indicated a one- (VSS for complexity one), three- (VSS for complexity two), and five-factor solution (Velicer MAP).

**Supplementary Fig. S1.** Scree plot showing the eigenvalues of the principal factors derived from the tetrachoric correlation matrix of HPS items


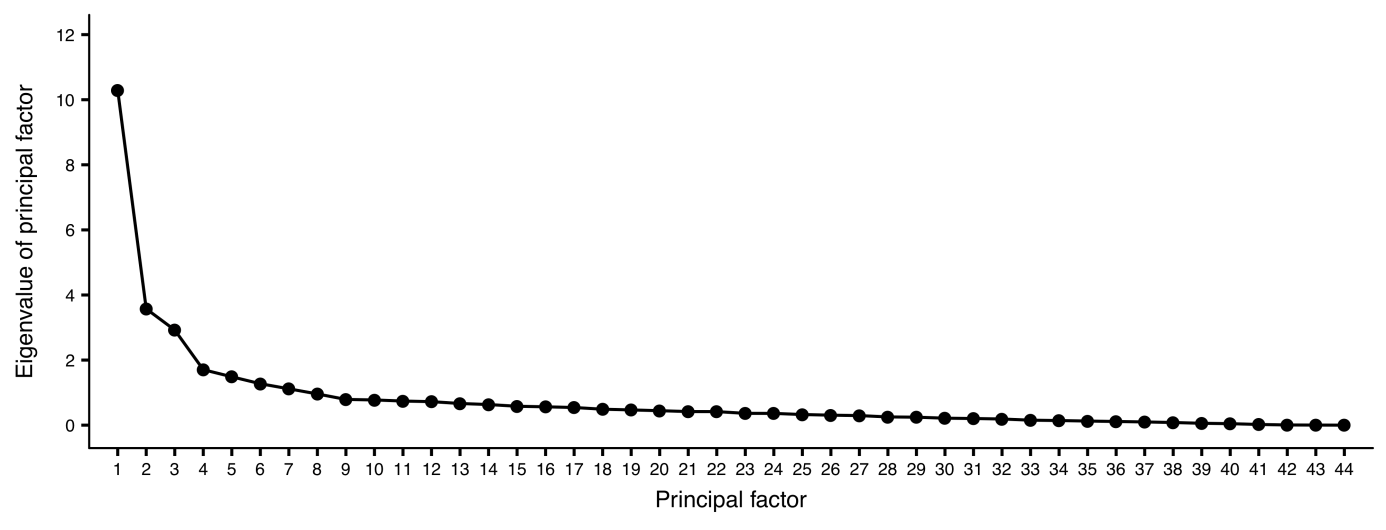


Eigenvalues are based on minimum residual factoring. Note that the eigenvalues of the 3-factor solution do not perfectly correspond to the shown solution.

The hierarchical cluster analysis stopped combinations at three clusters (see Figure S2). As we achieved the highest agreement across the abovementioned indices with the three-factor structure, we decided to proceed with the three-factor model. The sums of squared loadings for the three factors derived from the minimum residual factor analysis were 9.87, 3.14, and 2.47. Following promax rotation, the sums of squared loadings were 7.62, 5.27, and 2.58, accounting for 35% of the variance observed in the 44 items.

Figure S3 shows the correlation matrix of the HPS-items which were derived from the factor analysis. Factors one and two were correlated (0.45), while factor three was largely independent from factors one (0.06) and two (-0.05). The congruency of the factor-cluster solution was high, particularly regarding the first factor (.93), while factors two (.80) and three (.71) showed lower congruency coefficients.

**Supplementary Fig. S2.** iCLUST output based on the tetrachoric correlation matrix of HPS items


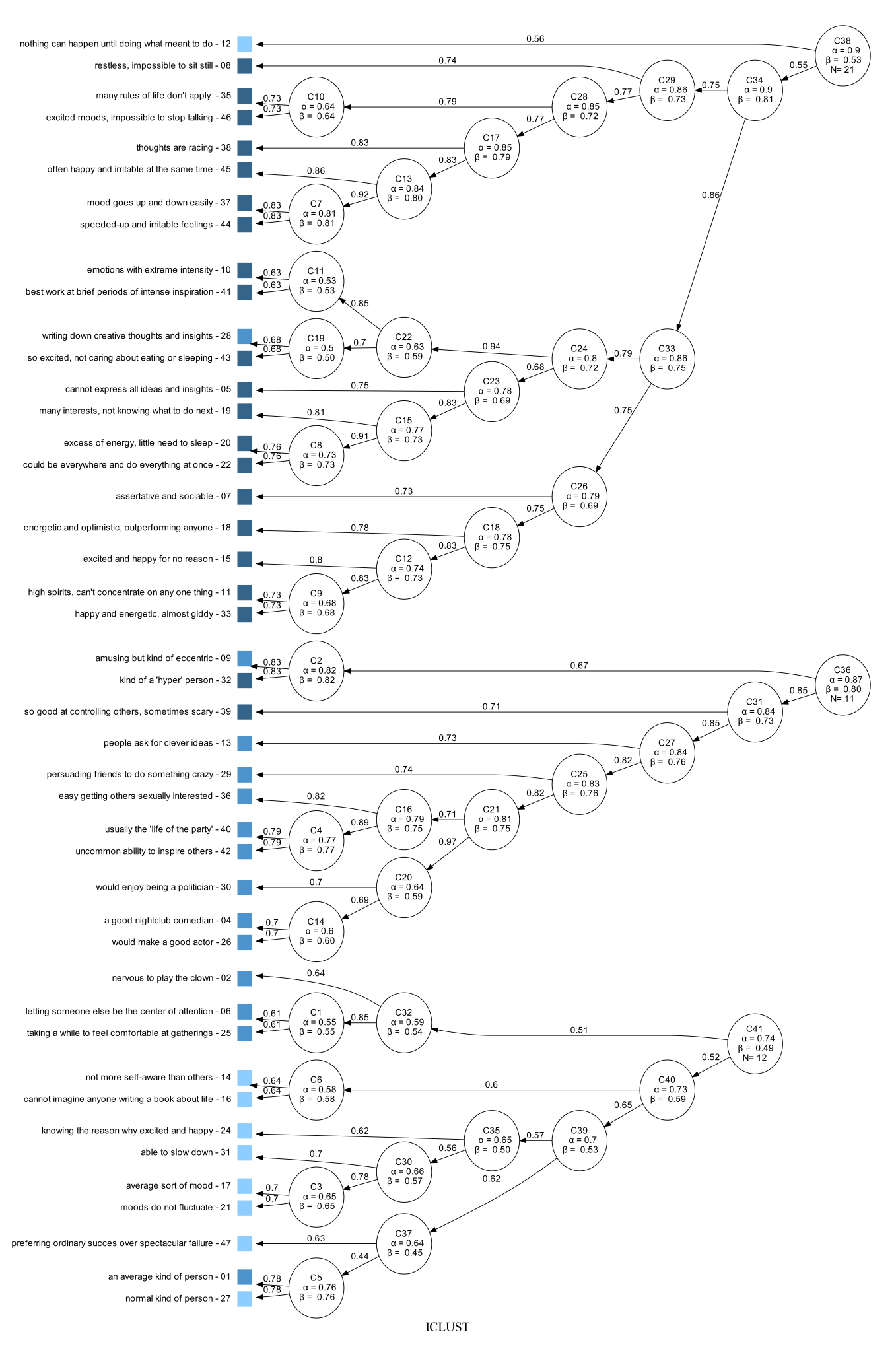


The two criteria for cluster combinations were as follows: (a) for two clusters of three or more items, combine only if the resulting cluster increases alpha past the maximum of the two subclusters, and (b) for two clusters of four items or more, combine only if the resulting cluster increases beta beyond the maximum of the two. Colored boxes indicate the congruency with factor analysis results, that is, colors indicate for which of the three factors hypomanic core, social vitality, and ordinariness (from dark to light blue) highest factor loadings were obtained.

**Supplementary Fig. S3.** Tetrachoric correlation matrix of the HPS items


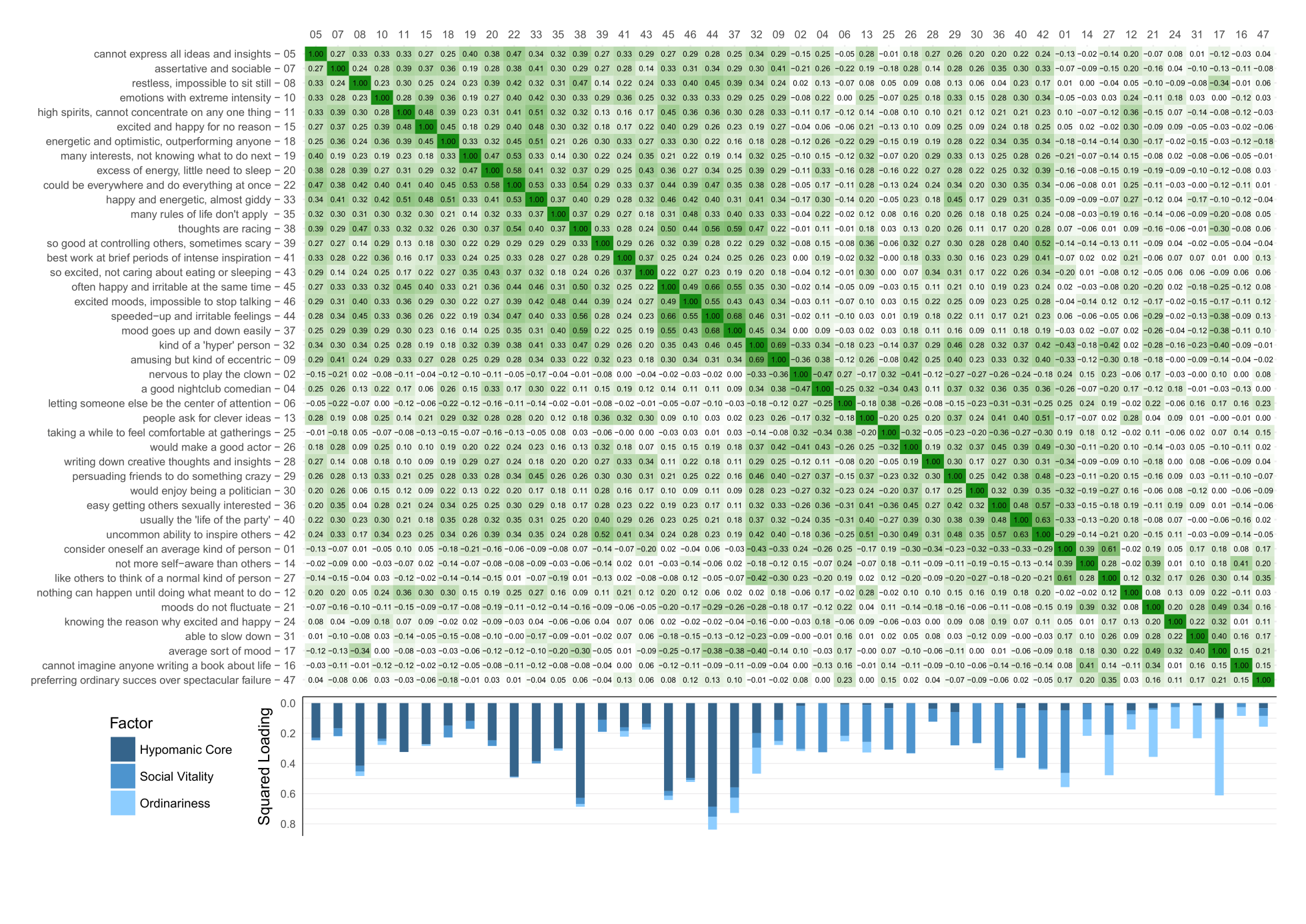


Squared factor loadings are shown for each item as blue shaded bars below the correlation matrix.

Supplementary Table S1 shows the loadings and IRT parameters of the HPS three-factor model and compares it to the factor solutions reported in two younger non-German samples by Rawlings et al.^4^ and Schalet et al.^5^

The first factor comprises items with clear hypomanic content and largely overlaps with the factor ‘moodiness’ by Rawlings et al.^4^ and the factors ‘mood volatility’ and ‘excitement’ by Schalet and colleagues^5^. Note that the term ‘mood volatility’ has been criticized as only very few items actually ask for the *lability* of subjects’ mood. Terrien et al.^6^ therefore named the first factor in their confirmatory factor analysis ‘hypomanic mood’*.* However, because the first factor also comprises cognitive and energetic aspects, we use the term *Hypomanic Core*.

Items loading high on the second factor describe high self-confidence, social dominance and leadership. This second factor largely overlaps with those previously labelled as ‘hypersociability’ and ‘social vitality’. In our study, we use the latter term, *Social Vitality*.

The third factor appears to reflect a characteristic that has previously been recognized as ‘ordinariness’, as it describes balanced and controlled people who describe themselves as average persons.^4^ Thus, we retain the term *Ordinariness*.

The factor score calculations for *Hypomanic Core, Social Vitality and Ordinariness* were based on those items showing factor discrimination parameters higher than or equal to the cut-off value 0.300.

**Supplementary Table S1.** Loadings and IRT parameters of the HPS three-factor model

|  |  | Loading | | |  | IRT discrimination parameter | | |  | IRT difficulty parameter | | |  |  |  |  |
| --- | --- | --- | --- | --- | --- | --- | --- | --- | --- | --- | --- | --- | --- | --- | --- | --- |
| Item |  | HYP | SOC | ORD |  | HYP | SOC | ORD |  | HYP | SOC | ORD |  | Rawlings et al. 2000 | Schalet et al. 2011 | Item text |
| 05 |  | **0.479** | 0.126 | 0.047 |  | **0.545** | 0.127 | 0.047 |  | **0.068** | 0.061 | 0.060 |  | Cognitive | Mood Volatility | Sometimes ideas and insights come to me so fast that I cannot express them all. |
| 07 |  | **0.408** | 0.227 | -0.053 |  | **0.447** | 0.233 | -0.053 |  | **0.954** | 0.894 | 0.872 |  | excluded | Social Vitality | In unfamiliar surroundings, I am often so assertive and sociable that I surprise myself . . . |
| 08 |  | **0.644** | -0.196 | -0.172 |  | **0.843** | -0.200 | -0.174 |  | **0.719** | 0.561 | 0.558 |  | Moodiness | Mood Volatility | There are often times when I am so restless that it is impossible for me to sit still. |
| 10 |  | **0.485** | 0.128 | 0.160 |  | **0.555** | 0.130 | 0.162 |  | **0.111** | 0.098 | 0.098 |  | Moodiness | Mood Volatility | When I feel an emotion, I usually feel it with extreme intensity. |
| 11 |  | **0.569** | -0.009 | -0.022 |  | **0.692** | -0.009 | -0.022 |  | **1.045** | 0.860 | 0.860 |  | Moodiness | Excitement | I am frequently in such high spirits that I can’t concentrate on any one thing for too . . . |
| 15 |  | **0.522** | 0.015 | 0.106 |  | **0.612** | 0.015 | 0.107 |  | **0.932** | 0.795 | 0.800 |  | Moodiness | Excitement | I often feel excited and happy for no apparent reason. |
| 18 |  | **0.386** | 0.279 | 0.045 |  | **0.418** | 0.291 | 0.045 |  | **1.170** | 1.124 | 1.081 |  | Moodiness | Excitement | I often have moods where I feel so energetic and optimistic that I feel I could . . . |
| 19 |  | **0.343** | 0.229 | 0.042 |  | **0.365** | 0.235 | 0.042 |  | **0.495** | 0.478 | 0.466 |  | Cognitive | Social Vitality | I have such a wide range of interests that I often don’t know what to do next. |
| 20 |  | **0.496** | 0.194 | -0.034 |  | **0.571** | 0.198 | -0.034 |  | **1.581** | 1.399 | 1.373 |  | Moodiness | Mood Volatility | There have often been times when I had such an excess of energy that I felt little . . . |
| 22 |  | **0.697** | 0.066 | 0.057 |  | **0.972** | 0.066 | 0.058 |  | **0.953** | 0.685 | 0.685 |  | Moodiness | Mood Volatility | I very frequently get into moods where I wish I could be everywhere and do everything . . . |
| 33 |  | **0.620** | 0.125 | 0.005 |  | **0.789** | 0.126 | 0.005 |  | **1.704** | 1.348 | 1.338 |  | Moodiness | Excitement | I often get so happy and energetic that I am almost giddy. |
| 35 |  | **0.548** | -0.010 | -0.120 |  | **0.654** | -0.010 | -0.121 |  | **1.257** | 1.052 | 1.059 |  | Moodiness | Mood Volatility | I often get into moods where I feel like many of the rules of life don’t apply to me. |
| 38 |  | **0.792** | -0.202 | -0.137 |  | **1.297** | -0.207 | -0.139 |  | **0.968** | 0.604 | 0.597 |  | Moodiness | Mood Volatility | I frequently find that my thoughts are racing. |
| 39 |  | **0.333** | 0.281 | 0.039 |  | **0.353** | 0.293 | 0.039 |  | **1.189** | 1.168 | 1.122 |  | excluded | Social Vitality | I am so good at controlling others that it sometimes scares me. |
| 41 |  | **0.400** | 0.159 | 0.193 |  | **0.437** | 0.161 | 0.197 |  | **0.269** | 0.250 | 0.251 |  | Cognitive | Mood Volatility | I do most of my best work during brief periods of intense inspiration. |
| 43 |  | **0.370** | 0.153 | 0.125 |  | **0.398** | 0.155 | 0.126 |  | **0.465** | 0.437 | 0.436 |  | Cognitive | Mood Volatility | I have often been so excited about an involving project that I didn’t care about eating . . . |
| 45 |  | **0.763** | -0.177 | -0.168 |  | **1.182** | -0.179 | -0.170 |  | **1.546** | 1.014 | 1.013 |  | Moodiness | Mood Volatility | I have often felt happy and irritable at the same time. |
| 46 |  | **0.704** | -0.118 | -0.114 |  | **0.992** | -0.118 | -0.115 |  | **1.992** | 1.424 | 1.424 |  | Moodiness | Excitement | I often get into excited moods where it’s almost impossible for me to stop talking. |
| 44 |  | **0.828** | -0.258 | -0.293 |  | **1.476** | -0.267 | **-0.307** |  | **1.851** | 1.074 | **1.086** |  | Moodiness | Mood Volatility | I frequently get into moods where I feel very speeded-up and irritable. |
| 37 |  | **0.747** | -0.262 | **-0.318** |  | **1.123** | -0.272 | **-0.335** |  | **1.038** | 0.715 | **0.728** |  | Moodiness | Mood Volatility | I seem to be a person whose mood goes up and down easily. |
| 32 |  | **0.445** | **0.312** | **-0.415** |  | **0.496** | **0.328** | **-0.456** |  | **1.789** | **1.686** | **1.761** |  | Hypersociability | Excitement | I am considered to be kind of a “hyper” person. |
| 09 |  | **0.334** | **0.374** | -0.162 |  | **0.355** | **0.403** | -0.164 |  | **0.996** | **1.012** | 0.951 |  | excluded | Mood Volatility | Many people consider me to be amusing but kind of eccentric. |
| 02 |  | 0.135 | **-0.535** | 0.113 |  | 0.137 | **-0.633** | 0.114 |  | -0.955 | **-1.120** | -0.953 |  | Hypersociability | Social Vitality | It would make me nervous to play the clown in front of other people. |
| 04 |  | 0.029 | **0.570** | 0.019 |  | 0.029 | **0.693** | 0.019 |  | 1.706 | **2.075** | 1.706 |  | Hypersociability | Social Vitality | I think I would make a good nightclub comedian. |
| 06 |  | 0.093 | **-0.456** | 0.192 |  | 0.094 | **-0.513** | 0.196 |  | -0.657 | **-0.735** | -0.666 |  | Hypersociability | Social Vitality | When with groups of people, I usually prefer to let someone else be the center . . . |
| 13 |  | 0.104 | **0.496** | 0.265 |  | 0.105 | **0.572** | 0.275 |  | -0.068 | **-0.078** | -0.070 |  | Cognitive | Social Vitality | People often come to me when they need a clever idea. |
| 25 |  | 0.182 | **-0.524** | 0.039 |  | 0.185 | **-0.615** | 0.039 |  | -0.731 | **-0.843** | -0.719 |  | Hypersociability | Social Vitality | When I go to a gathering where I don’t know anyone, it usually takes me a while . . . |
| 26 |  | 0.042 | **0.574** | -0.049 |  | 0.042 | **0.702** | -0.049 |  | 0.954 | **1.165** | 0.954 |  | excluded | Social Vitality | I think I would make a good actor, because I can play many roles convincingly. |
| 28 |  | 0.193 | 0.293 | 0.017 |  | 0.196 | **0.306** | 0.017 |  | 1.155 | **1.185** | 1.133 |  | Cognitive | Excluded | I frequently write down the thoughts and insights that come to me when I am thinking . . . |
| 29 |  | 0.244 | **0.469** | 0.031 |  | 0.252 | **0.531** | 0.031 |  | 1.196 | **1.313** | 1.161 |  | Hypersociability | Social Vitality | I have often persuaded groups of friends to do something really adventurous or crazy. |
| 30 |  | 0.005 | **0.515** | -0.027 |  | 0.005 | **0.601** | -0.027 |  | 1.282 | **1.495** | 1.282 |  | excluded | Social Vitality | I would really enjoy being a politician and hitting the campaign trail. |
| 36 |  | 0.073 | **0.652** | 0.120 |  | 0.073 | **0.859** | 0.120 |  | 0.518 | **0.681** | 0.520 |  | excluded | Social Vitality | I find it easy to get others to become sexually interested in me.^a^ |
| 40 |  | 0.181 | **0.573** | 0.056 |  | 0.184 | **0.699** | 0.056 |  | 1.181 | **1.417** | 1.164 |  | Hypersociability | Social Vitality | At social gatherings, I am usually the “life of the party.” |
| 42 |  | 0.218 | **0.621** | 0.086 |  | 0.224 | **0.793** | 0.086 |  | 0.990 | **1.232** | 0.969 |  | Cognitive | Social Vitality | I seem to have an uncommon ability to persuade and inspire others. |
| 01 |  | 0.218 | **-0.644** | **0.307** |  | 0.224 | **-0.842** | **0.323** |  | -1.516 | **-1.934** | **-1.554** |  | Ordinariness | Social Vitality | I consider myself to be pretty much an average kind of person. |
| 14 |  | 0.079 | **-0.319** | **0.330** |  | 0.080 | **-0.337** | **0.349** |  | -0.973 | **-1.024** | **-1.027** |  | Ordinariness | Social Vitality | I am no more self-aware than the majority of people. |
| 27 |  | 0.123 | **-0.442** | **0.517** |  | 0.124 | **-0.492** | **0.605** |  | -2.003 | **-2.216** | **-2.323** |  | Ordinariness | Social Vitality | I like to have others think of me as a normal kind of person. |
| 12 |  | 0.218 | 0.164 | **0.317** |  | 0.223 | 0.166 | **0.334** |  | -0.383 | -0.379 | **-0.394** |  | Cognitive | Excluded | I sometimes have felt that nothing can happen to me until I do what I am meant to . . . |
| 21 |  | -0.180 | -0.107 | **0.559** |  | -0.184 | -0.108 | **0.674** |  | -1.243 | -1.229 | **-1.474** |  | Moodiness | Mood Volatility | My moods do not seem to fluctuate any more than most people’s do. |
| 24 |  | -0.057 | 0.154 | **0.377** |  | -0.057 | 0.155 | **0.407** |  | -1.577 | -1.594 | **-1.700** |  | Moodiness | Excluded | When I feel very excited and happy, I almost always know the reason why. |
| 31 |  | -0.126 | 0.030 | **0.465** |  | -0.127 | 0.030 | **0.526** |  | -2.023 | -2.008 | **-2.267** |  | Moodiness | Mood Volatility | I can usually slow myself down when I want to. |
| 17 |  | **-0.314** | 0.100 | **0.709** |  | **-0.330** | 0.101 | **1.006** |  | **-1.609** | -1.536 | **-2.168** |  | excluded | Excitement | I am usually in an average sort of mood, not too high and not too low. |
| 16 |  | -0.050 | -0.156 | 0.241 |  | -0.050 | -0.158 | 0.248 |  | -0.431 | -0.436 | -0.443 |  | Ordinariness | Social Vitality | I can’t imagine that anyone would ever write a book about my life. |
| 47 |  | 0.181 | -0.228 | 0.268 |  | 0.184 | -0.234 | 0.278 |  | -0.891 | -0.900 | -0.909 |  | Ordinariness | Social Vitality | I would rather be an ordinary success in life than a spectacular failure. |

HPS: Hypomanic Personality Scale (Eckblad & Chapman, 1986), IRT: Item response theory, HYP: HPS facet ‘hypomanic core‘, SOC: HPS facet ‘social vitality’, ORD: HPS facet ‘ordinariness’, ^a^ No sexual content in the German translation^1^ of the item. Minimum residual factor analysis was based on tetrachoric correlations. Loadings and IRT discrimination parameters are bold where they are higher than or equal to the cut-off value 0.300. IRT difficulty parameters are bold where the corresponding IRT discrimination parameters are higher than or equal to the cut-off value 0.300. Note that subsequent factor score calculations were based on those items showing factor discrimination parameters higher than or equal to the cut-off value 0.300.

**Correlation analyses**

Extending from meta-analyses’ findings showing various forms of sleep impairment in euthymic BD^7,8^, the current study examined the association of individual sleep variables with the HPS, a psychometric risk factor for BD. Because each test was hypothesis-driven, there was no need for multiple-testing correction^9,10^. However, to confirm that results persisted after correction for multiple testing, we additionally calculated the False Discovery Rate (FDR)-corrected *p* values according to Benjamini-Hochberg^11^ and regarded associations with FDR < 0.05 as significant after multiple testing correction. In total, 40 out of the 46 nominal significant correlations remained significant after multiple-testing correction (see Table S2).

**Supplementary Table S2.** Partial Spearman correlations between HPS and sleep-wake variables. FDR-corrected and nominal p-values are given for comparison

|  | HPS total  sum-score | | | |  | HPS subscale Hypomanic Core | | | |  | HPS subscale Social Vitality | | | |  | HPS subscale Ordinariness | | | |
| --- | --- | --- | --- | --- | --- | --- | --- | --- | --- | --- | --- | --- | --- | --- | --- | --- | --- | --- | --- |
|  | rho | *p* | FDR |  |  | rho | *p* | FDR |  |  | rho | *p* | FDR |  |  | rho | *p* | FDR |  |
| **Actigraphy** (n = 771) |  |  |  |  |  |  |  |  |  |  |  |  |  |  |  |  |  |  |  |
| **Means** |  |  |  |  |  |  |  |  |  |  |  |  |  |  |  |  |  |  |  |
| Sleep-onset latency | .038 | .294 | .399 |  |  | .042 | .240 | .342 |  |  | .054 | .134 | .200 |  |  | -.035 | .329 | .432 |  |
| Sleep-onset time | -.006 | .864 | .907 |  |  | -.035 | .329 | .432 |  |  | .025 | .497 | .580 |  |  | .061 | .089 | .144 |  |
| Sleep-offset time | -.013 | .721 | .797 |  |  | -.041 | .252 | .353 |  |  | .007 | .851 | .905 |  |  | .056 | .118 | .180 |  |
| Sleep duration | -.079 | .029 | .057 | ^*^ |  | -.072 | .046 | .084 | ^*^ |  | -.059 | .101 | .160 |  |  | .034 | .350 | .439 |  |
| NWAK | .078 | .030 | .058 | ^*^ |  | .069 | .056 | .098 |  |  | .069 | .056 | .098 |  |  | -.011 | .757 | .826 |  |
| WASO | .103 | .004 | .014 | ^**^ |  | .091 | .011 | .025 | ^**^ |  | .080 | .027 | .055 | ^*^ |  | -.046 | .203 | .299 |  |
| Sleep efficiency | -.106 | .003 | .011 | ^**^ |  | -.101 | .005 | .016 | ^**^ |  | -.086 | .017 | .035 | ^**^ |  | .033 | .361 | .446 |  |
| **Night-to-night variability** |  |  |  |  |  |  |  |  |  |  |  |  |  |  |  |  |  |  |  |
| Sleep-onset latency | .019 | .594 | .665 |  |  | -.005 | .893 | .926 |  |  | .076 | .036 | .067 | ^*^ |  | -.026 | .470 | .556 |  |
| Sleep-onset time | .122 | 7E-4 | .005 | ^**^ |  | .102 | .004 | .014 | ^**^ |  | .113 | .002 | .010 | ^**^ |  | .009 | .813 | .876 |  |
| Sleep-offset time | .110 | .002 | .010 | ^**^ |  | .119 | 9E-4 | .006 | ^**^ |  | .069 | .057 | .098 |  |  | -.062 | .087 | .144 |  |
| Sleep duration | .098 | .006 | .018 | ^**^ |  | .092 | .011 | .025 | ^**^ |  | .097 | .007 | .019 | ^**^ |  | -.003 | .931 | .943 |  |
| NWAK | .145 | 6E-5 | 9E-4 | ^**^ |  | .109 | .002 | .010 | ^**^ |  | .152 | 2E-5 | 7E-4 | ^**^ |  | -.092 | .011 | .025 | ^**^ |
| WASO | .115 | .001 | .007 | ^**^ |  | .116 | .001 | .007 | ^**^ |  | .095 | .008 | .020 | ^**^ |  | -.109 | .002 | .010 | ^**^ |
| Sleep efficiency | .106 | .003 | .011 | ^**^ |  | .095 | .008 | .020 | ^**^ |  | .103 | .004 | .014 | ^**^ |  | -.092 | .011 | .025 | ^**^ |
| **PSQI** (n = 1766) |  |  |  |  |  |  |  |  |  |  |  |  |  |  |  |  |  |  |  |
| Sleep-onset latency^a^ | .013 | .576 | .660 |  |  | .078 | .001 | .007 | ^**^ |  | -.080 | 8E-4 | .005 | ^**^ |  | -.098 | 4E-5 | 8E-4 | ^**^ |
| Bedtime^a,b^ | .029 | .230 | .333 |  |  | .018 | .444 | .533 |  |  | .041 | .087 | .144 |  |  | .001 | .968 | .968 |  |
| Get-up time^a^ | -.022 | .348 | .439 |  |  | -.023 | .344 | .439 |  |  | -.020 | .413 | .502 |  |  | -.013 | .581 | .660 |  |
| Sleep duration^a^ | -.038 | .114 | .177 |  |  | -.095 | 7E-5 | 9E-4 | ^**^ |  | .027 | .261 | .359 |  |  | .081 | 6E-4 | .005 | ^**^ |
| Sleep efficiency^c^ | .002 | .925 | .943 |  |  | -.065 | .006 | .018 | ^**^ |  | .071 | .003 | .011 | ^**^ |  | .091 | 1E-4 | .002 | ^**^ |
| Daytime sleepiness^a^ | .075 | .002 | .010 | ^**^ |  | .088 | 2E-4 | .002 | ^**^ |  | .052 | .028 | .057 | ^*^ |  | -.086 | 3E-4 | .003 | ^**^ |
| PSQI score^d^ | .063 | .008 | .020 | ^**^ |  | .158 | 3E-11 | 1E-9 | ^**^ |  | -.063 | .008 | .020 | ^**^ |  | -.165 | 3E-12 | 3E-10 | ^**^ |
| Night-to-night variability is operationalized by intraindividual standard deviation (ISD) across a single subject's multiple nights.  Note that *Hypomanic Core, Social Vitality* and *Ordinariness* here refer to factor scores derived from factor analyses. Results were additionally confirmed by analyses with traditional sum scores (see Supplementary Table 4).  Effects of sex and age were partialled out. FDR: False Discovery Rate according to Benjamini and Hochberg^11^; NWAK: Number of awakenings; WASO: wake after sleep-onset time  ^*^ *p* < .05 (two-sided nominal significance)  ^**^ FDR < .05 (*p* value corrected for all tested associations applying the Benjamini-Hochberg FDR method^11^)  ^a^ based on the respective PSQI item ^b^ time subject goes to bed; ^c^ quotient of sleep duration and time in bed with the latter calculated from bedtime and get-up time; ^d^ PSQI total score calculated according the manual from all PSQI components; higher PSQI scores mean worse sleep quality. | | | | | | | | | | | | | | | | | | | |

**Supplementary Table S3.** Comparison of Spearman correlations

|  | | | | | | | | | | | | | | | | | |  |
| --- | --- | --- | --- | --- | --- | --- | --- | --- | --- | --- | --- | --- | --- | --- | --- | --- | --- | --- |
|  | Spearman’s rho | | | |  | P value of Spearman’s rho comparisons | | | | | | | | | | | |  |
|  | HT | HC | SV | OD |  | HT vs.  HC | | HT vs.  SV | | HT vs.  OD | | HC vs.  SV | | HC vs.  OD | | SV vs.  OD | | |
| **Actigraphy** (n = 771) |  |  |  |  |  |  |  |  |  |  |  |  |  |  |  |  |  | |
| **Means** |  |  |  |  |  |  |  |  |  |  |  |  |  |  |  |  |  | |
| Sleep-onset latency | .038 | .042 | .054 | -.035 |  | .831 |  | .549 |  | .211 |  | .779 |  | .180 |  | .112 |  | |
| Sleep-onset time | -.006 | -.035 | .025 | .061 |  | .172 |  | .258 |  | .247 |  | .153 |  | .095 |  | .512 |  | |
| Sleep-offset time | -.013 | -.041 | .007 | .056 |  | .181 |  | .469 |  | .235 |  | .249 |  | .091 |  | .377 |  | |
| Sleep duration | -.079 | -.072 | -.059 | .034 |  | .750 |  | .471 |  | .054 |  | .759 |  | .068 |  | .098 |  | |
| NWAK | .078 | .069 | .069 | -.011 |  | .667 |  | .734 |  | .125 |  | .999 |  | .165 |  | .153 |  | |
| WASO | .103 | .091 | .080 | -.046 |  | .593 |  | .399 |  | .011 | ** | .782 |  | .018 | * | .025 | * | |
| Sleep efficiency | -.106 | -.101 | -.086 | .033 |  | .797 |  | .467 |  | .017 | * | .732 |  | .021 | * | .033 | * | |
| **Night-to-night variability** |  |  |  |  |  |  |  |  |  |  |  |  |  |  |  |  |  | |
| Sleep-onset latency | .019 | -.005 | .076 | -.026 |  | .257 |  | .038 | * | .437 |  | .054 |  | .714 |  | .070 |  | |
| Sleep-onset time | .122 | .102 | .113 | .009 |  | .357 |  | .727 |  | .051 |  | .808 |  | .104 |  | .063 |  | |
| Sleep-offset time | .110 | .119 | .069 | -.062 |  | .680 |  | .124 |  | .003 | ** | .226 |  | .002 | ** | .020 | * | |
| Sleep duration | .098 | .092 | .097 | -.003 |  | .748 |  | .963 |  | .081 |  | .894 |  | .101 |  | .074 |  | |
| NWAK | .145 | .109 | .152 | -.092 |  | .092 |  | .787 |  | 5E-5 | ** | .301 |  | 5E-4 | ** | 1E-5 | ** | |
| WASO | .115 | .116 | .095 | -.109 |  | .963 |  | .463 |  | 1E-4 | ** | .616 |  | 9E-5 | ** | 3E-4 | ** | |
| Sleep efficiency | .106 | .095 | .103 | -.092 |  | .599 |  | .923 |  | 7E-4 | ** | .838 |  | .001 | ** | 5E-4 | ** | |
| **PSQI** (n = 1766) |  |  |  |  |  |  |  |  |  |  |  |  |  |  |  |  |  | |
| Sleep-onset latency^a^ | .013 | .078 | -.080 | -.098 |  | 2E-6 | ** | 1E-7 | ** | .004 | ** | 5E-9 | ** | 4E-6 | ** | .624 |  | |
| Bedtime^a,b^ | .029 | .018 | .041 | .001 |  | .450 |  | .493 |  | .470 |  | .408 |  | .653 |  | .273 |  | |
| Get-up time^a^ | -.022 | -.023 | -.020 | -.013 |  | .989 |  | .872 |  | .810 |  | .911 |  | .806 |  | .860 |  | |
| Sleep duration^a^ | -.038 | -.095 | .027 | .081 |  | 3E-5 | ** | 3E-4 | ** | .002 | ** | 7E-6 | ** | 4E-6 | ** | .132 |  | |
| Sleep efficiency^c^ | .002 | -.065 | .071 | .091 |  | 1E-6 | ** | 1E-4 | ** | .021 | * | 6E-7 | ** | 5E-5 | ** | .586 |  | |
| Daytime sleepiness^a^ | .075 | .088 | .052 | -.086 |  | .315 |  | .202 |  | 3E-5 | ** | .181 |  | 5E-6 | ** | 1E-4 | ** | |
| PSQI score^d^ | .063 | .158 | -.063 | -.165 |  | 5E-12 | ** | 7E-13 | ** | 2E-9 | ** | 2E-16 | ** | 2E-17 | ** | .004 | ** | |
| Comparisons of Spearman’s rho correlation coefficients was carried out according to the formula by Dunn & Clark^12^ as implemented in R package cocor v.1.1-3^13^.  HT *HPS Total*; HC *Hypomanic Core*; SV *Social Vitality*; OD *Ordinariness*  * *p* < .05 (two-sided nominal significance)  ** FDR < .05 (*p* value corrected for all tested associations using Benjamini-Hochberg FDR method)  Night-to-night variability is operationalized by intraindividual standard deviation (ISD) across a single subject's multiple nights.  ^a^ based on the respective PSQI item ^b^ time subject goes to bed; ^c^ quotient of sleep duration and time in bed with the latter calculated from bedtime and get-up time; ^d^ PSQI total score calculated according the manual from all PSQI components; higher PSQI scores mean worse sleep quality. | | | | | | | | | | | | | | | | | |  |

**Secondary analyses for confirmation of results**

Further analyses were conducted to confirm the correlational results. First, all correlational analyses were repeated with unweighted sum scores for each HPS subscale instead of factor scores, which resulted in comparable, albeit, as expected, somewhat weaker associations (see Table S1). In total, 39 out of 84 correlations reached the level of nominal significance. Of those, 32 remained significant after multiple-testing correction. A reduction in the number of significant associations was most pronounced for the facet *Ordinariness*.

**Supplementary Table S4.** Partial Spearman correlations between HPS (unweighted sum scores) and the sleep-wake variables

|  | | | | | | | | | | | | | | | | | | | |
| --- | --- | --- | --- | --- | --- | --- | --- | --- | --- | --- | --- | --- | --- | --- | --- | --- | --- | --- | --- |
|  | HPS sum score | | | |  | Unweighted sum score of Hypomanic Core | | | |  | Unweighted sum score of Social Vitality | | | |  | Unweighted sum score of Ordinariness | | | |
|  | rho | *p* | FDR |  |  | rho | *p* | FDR |  |  | rho | *p* | FDR |  |  | rho | *p* | FDR |  |
| **Actigraphy** (n = 771) |  |  |  |  |  |  |  |  |  |  |  |  |  |  |  |  |  |  |  |
| **Means** |  |  |  |  |  |  |  |  |  |  |  |  |  |  |  |  |  |  |  |
| Sleep-onset latency | .038 | .294 | .419 |  |  | .032 | .372 | .480 |  |  | .056 | .124 | .200 |  |  | -.013 | .719 | .784 |  |
| Sleep-onset time | -.006 | .864 | .874 |  |  | -.008 | .816 | .836 |  |  | .044 | .228 | .339 |  |  | .033 | .356 | .475 |  |
| Sleep-offset time | -.013 | .721 | .784 |  |  | -.024 | .508 | .611 |  |  | .013 | .728 | .784 |  |  | .045 | .214 | .326 |  |
| Sleep duration | -.079 | .029 | .068 | * |  | -.079 | .029 | .068 | * |  | -.063 | .083 | .151 |  |  | .024 | .509 | .611 |  |
| NWAK | .078 | .030 | .068 | * |  | .063 | .080 | .150 |  |  | .059 | .101 | .170 |  |  | .027 | .456 | .563 |  |
| WASO | .103 | .004 | .020 | ** |  | .085 | .018 | .047 | ** |  | .069 | .056 | .110 |  |  | .009 | .812 | .836 |  |
| Sleep efficiency | -.106 | .003 | .015 | ** |  | -.094 | .009 | .030 | ** |  | -.079 | .029 | .068 | * |  | -.010 | .775 | .818 |  |
| **Night-to-night variability** |  |  |  |  |  |  |  |  |  |  |  |  |  |  |  |  |  |  |  |
| Sleep-onset latency | .019 | .594 | .674 |  |  | -.010 | .779 | .818 |  |  | .075 | .038 | .083 | * |  | -.033 | .366 | .480 |  |
| Sleep-onset time | .122 | 7E-4 | .007 | ** |  | .098 | .006 | .025 | ** |  | .123 | 7E-4 | .007 | ** |  | .077 | .033 | .073 | * |
| Sleep-offset time | .110 | .002 | .012 | ** |  | .125 | 5E-4 | .007 | ** |  | .059 | .100 | .170 |  |  | -.028 | .436 | .547 |  |
| Sleep duration | .098 | .006 | .025 | ** |  | .087 | .015 | .043 | ** |  | .097 | .007 | .027 | ** |  | .061 | .093 | .167 |  |
| NWAK | .145 | 6E-5 | .002 | ** |  | .115 | .001 | .010 | ** |  | .145 | 6E-5 | .002 | ** |  | -.022 | .535 | .633 |  |
| WASO | .115 | .001 | .010 | ** |  | .107 | .003 | .015 | ** |  | .082 | .024 | .060 | * |  | -.047 | .193 | .304 |  |
| Sleep efficiency | .106 | .003 | .015 | ** |  | .090 | .013 | .037 | ** |  | .094 | .009 | .030 | ** |  | -.036 | .315 | .434 |  |
| **PSQI** (n = 1766) |  |  |  |  |  |  |  |  |  |  |  |  |  |  |  |  |  |  |  |
| Sleep-onset latency^a^ | .013 | .576 | .663 |  |  | .063 | .008 | .029 | ** |  | -.079 | 8E-4 | .007 | ** |  | -.057 | .017 | .045 | ** |
| Bedtime^a,b^ | .029 | .230 | .339 |  |  | .031 | .196 | .304 |  |  | .046 | .051 | .105 |  |  | .009 | .699 | .783 |  |
| Get-up time^a^ | -.022 | .348 | .471 |  |  | -.025 | .299 | .419 |  |  | -.019 | .432 | .547 |  |  | -.013 | .573 | .663 |  |
| Sleep duration^a^ | -.038 | .114 | .187 |  |  | -.088 | 2E-4 | .004 | ** |  | .028 | .236 | .342 |  |  | .040 | .096 | .168 |  |
| Sleep efficiency^c^ | .002 | .925 | .925 |  |  | -.046 | .053 | .105 |  |  | .073 | .002 | .012 | ** |  | .046 | .051 | .105 |  |
| Daytime sleepiness^a^ | .075 | .002 | .012 | ** |  | .081 | 6E-4 | .007 | ** |  | .061 | .010 | .031 | ** |  | -.042 | .080 | .150 |  |
| PSQI score^d^ | .063 | .008 | .029 | ** |  | .127 | 9E-8 | 7E-6 | ** |  | -.061 | .011 | .032 | ** |  | -.088 | 2E-4 | .004 | ** |
| Effects of sex and age were partialled out. FDR: False Discovery Rate according to Benjamini and Hochberg^11^; NWAK: Number of awakenings; WASO: wake after sleep-onset time  * *p* < .05 (two-sided nominal significance)  ** FDR < .05 (*p* value corrected for all tested associations applying the Benjamini-Hochberg FDR method^11^)  Night-to-night variability is operationalized by intraindividual standard deviation (ISD) across a single subject's multiple nights.  ^a^ based on the respective PSQI item ^b^ time subject goes to bed; ^c^ quotient of sleep duration and time in bed with the latter calculated from bedtime and get-up time; ^d^ PSQI total score calculated according the manual from all PSQI components; higher PSQI scores mean worse sleep quality. | | | | | | | | | | | | | | | | | | | |

As another confirmation of results, analyses were repeated using HPS decile extreme groups (as is often done in psychometric high-risk studies). HPS extreme groups were defined as the lower (HPS-); and upper (HPS+) decile of the distribution (HPS- : n=61, range 0-2, mean sum score = 1.39, SD=0.74; HPS +: n=63, mean sum score = 20.23, SD = 3.79, range: 16-37).

Results from extreme group comparisons are presented in Supplementary Table S5 and illustrated in Supplementary Figure S4.

In total, 38 out of 84 comparisons reached the level of nominal significance, of which 23 remained significant after multiple-testing correction. Concerning objective sleep, HPS high-scorers had significantly lower sleep efficiency and more time WASO. In accordance with the correlational analyses, the extreme groups more strongly differed concerning the night-to-night variability of sleep parameters than concerning the mean sleep parameters. The HPS high scorers showed greater variability in all sleep variables, except for of sleep-onset latency which did not reach significance level. Also in line with correlation analyses, HPS high-scorers had lower self-reported sleep efficiency, worse overall sleep quality (PSQI total score) and greater daytime sleepiness.

**Supplementary Table S5**. HPS total sum score and sleep-wake variables – extreme group comparisons

|  | | | | | | | | | |  |  |
| --- | --- | --- | --- | --- | --- | --- | --- | --- | --- | --- | --- |
|  |  |  |  | Inference statistics | | | | | |  |  |
|  | HPS+ | HPS- |  | Mean ranks^a^ | χ² | η² | *p* | FDR |  |  |  |
| *N* | 63 | 61 |  |  |  |  |  |  |  |  |  |
| **Demography** |  |  |  |  |  |  |  |  |  |  |  |
| Sex (f/m) | 28 / 35 | 27 / 34 |  |  | 0.000 | < 0.001 | .984 |  |  |  |  |
| Age (yrs) | 70.2 (4.1) | 70.1 (4.2) |  | 63.12 / 61.86 | 0.038 | < 0.001 | .845 |  |  |  |  |
| **Actigraphy** |  |  |  |  |  |  |  |  |  |  |  |
| **Means** |  |  |  |  |  |  |  |  |  |  |  |
| Sleep-onset latency (min) | 0:09 (0:06) | 0:07 (0:04) |  | 68.13 / 56.69 | 3.140 | 0.025 | .076 | .140 |  |  |  |
| Sleep-onset time (h:min) | 23:29 (0:51) | 23:29 (0:49) |  | 61.58 / 63.45 | 0.084 | 0.001 | .772 | .809 |  |  |  |
| Sleep-offset time (h:min) | 7:00 (0:43) | 7:04 (0:44) |  | 62.06 / 62.96 | 0.020 | < 0.001 | .889 | .889 |  |  |  |
| Sleep duration (h:min) | 6:12 (1:15) | 6:34 (0:53) |  | 58.78 / 66.34 | 1.374 | 0.011 | .241 | .332 |  |  |  |
| NWAK | 3.23 (1.2) | 2.92 (1.2) |  | 66.30 / 58.57 | 1.436 | 0.012 | .231 | .332 |  |  |  |
| WASO (h:min) | 1:19 (0:39) | 1:00 (0:29) |  | 70.83 / 53.90 | 6.872 | 0.055 | .009 | .021 | ** |  |  |
| Sleep efficiency (%) | 81.8 (6.3) | 85.3 (6.2) |  | 53.05 / 72.26 | 8.859 | 0.071 | .003 | .011 | ** |  |  |
| **Night-to-night variability** |  |  |  |  |  |  |  |  |  |  |  |
| Sleep-onset latency (min) | 0:07 (0:04) | 0:06 (0:03) |  | 65.84 / 59.05 | 1.107 | 0.009 | .293 | .379 |  |  |  |
| Sleep-onset time (h:min) | 0:46 (0:24) | 0:35 (0:19) |  | 70.13 / 54.62 | 5.768 | 0.047 | .016 | .036 | ** |  |  |
| Sleep-offset time (h:min) | 0:52 (0:35) | 0:37 (0:18) |  | 73.52 / 51.11 | 12.049 | 0.097 | 5E-4 | .003 | ** |  |  |
| Sleep duration (h:min) | 0:59 (0:20) | 0:46 (0:18) |  | 73.83 / 50.80 | 12.717 | 0.103 | 4E-4 | .003 | ** |  |  |
| NWAK | 1.62 (0.5) | 1.29 (0.5) |  | 74.49 / 50.11 | 14.267 | 0.115 | 2E-4 | .003 | ** |  |  |
| WASO (h:min) | 0:39 (0:21) | 0:27 (0:14) |  | 73.32 / 51.33 | 11.602 | 0.094 | 7E-4 | .003 | ** |  |  |
| Sleep efficiency (%) | 6.73 (2.6) | 5.50 (2.2) |  | 71.14 / 53.57 | 7.406 | 0.060 | .006 | .018 | ** |  |  |
| **PSQI** |  |  |  |  |  |  |  |  |  |  |  |
| Sleep-onset latency^b^ | 0:24 (0:26) | 0:16 (0:14) |  | 66.31 / 58.57 | 1.485 | 0.012 | .223 | .332 |  |  |  |
| Bedtime^b,c^ | 23:00 (0:52) | 22:52 (0:46) |  | 65.34 / 59.57 | 0.841 | 0.007 | .359 | .439 |  |  |  |
| Get-up time^b^ | 7:24 (0:45) | 7:16 (0:42) |  | 64.79 / 60.13 | 0.545 | 0.004 | .460 | .533 |  |  |  |
| Sleep duration^b^ | 6:51 (1:05) | 7:09 (1:03) |  | 57.54 / 67.62 | 2.714 | 0.022 | .099 | .168 |  |  |  |
| Sleep efficiency^d^ | 82.1 (11.9) | 85.5 (10.6) |  | 56.60 / 68.60 | 3.475 | 0.028 | .062 | .125 |  |  |  |
| Daytime sleepiness^b^ | 0.30 (0.64) | 0.07 (0.31) |  | 67.77 / 57.06 | 7.722 | 0.062 | .005 | .017 | ** |  |  |
| PSQI score^e^ | 5.87 (3.2) | 3.92 (2.9) |  | 74.05 / 50.57 | 13.444 | 0.108 | 2E-4 | .003 | ** |  |  |
| Except for sex and *n*, descriptive statistics are presented as mean (standard deviation). Inference statistics are based on χ²-Test (sex), or Kruskal-Wallis Test (all other variables). All χ² values are specified by one degree of freedom. The effect size η² was calculated by squaring r derived from r=√(χ²/N) according to Rosenthal & DiMatteo^14^. FDR: False Discovery Rate according to Benjamini and Hochberg^11^  * *p* < .05 (two-sided nominal significance)  ** FDR < .05 (*p* value corrected for all shown associations applying Benjamini-Hochberg FDR method^11^)  Night-to-night variability is operationalized by intraindividual standard deviation (ISD) across a single subject's multiple nights.  ^a^ Mean rank HPS+ / mean rank HPS-  ^b^ based on the respective PSQI item ^c^ time subject goes to bed; ^d^ quotient of sleep duration and time in bed with the latter calculated from bedtime and get-up time; ^e^ PSQI total score calculated according the manual from all PSQI components; higher PSQI scores mean worse sleep quality. | | | | | | | | | |  |  |
|  |  |  |  |  |  |  |  |  |  |  |  |
|  |  |  |  |  |  |  |  |  |  |  |  |

**Supplementary Fig. S4.** Boxplots of actigraphy and PSQI variables stratified by HPS extreme groups

**
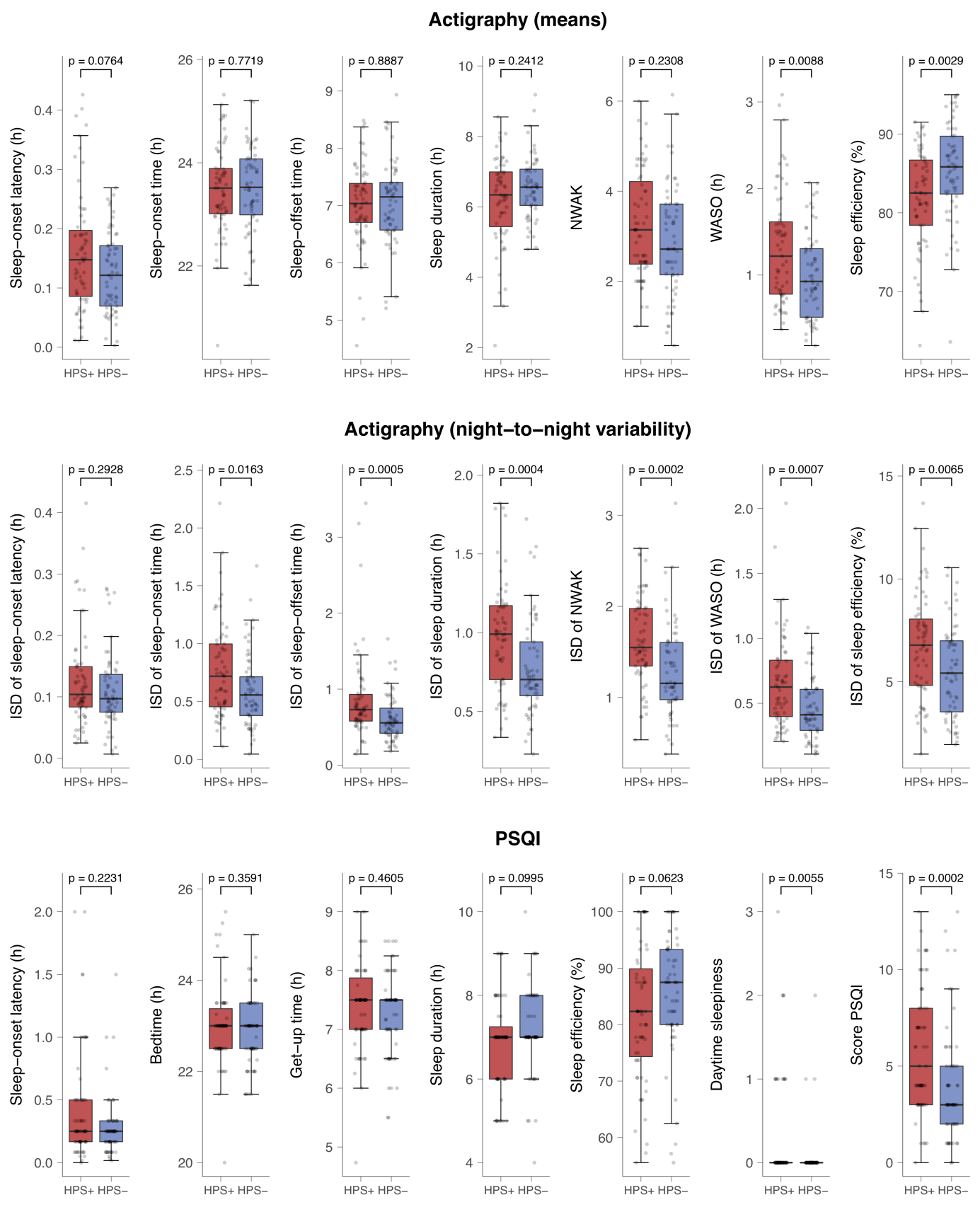
**

Intraindividual night-to-night variability is operationalized by intraindividual standard deviation (ISD) across a single subject's multiple nights. Boxplots are stratified by top and bottom decile HPS groups (HPS+, N=63 vs. HPS-, N=61). Boxes represent the interquartile range of each distribution (data between the lower and upper quartile), with the horizontal line corresponding to the median. Whiskers extend to the furthest observation within 1.5 times the interquartile range from the lower and upper quartile. Dots represent single data points, jittered horizontally to avoid overplotting. Note that statistical analyses were based on nonparametric (ranked) data.

**Exploratory regression analysis with HPS subscales**

In the present study, lowest type-I-error probabilities were obtained for associations between the PSQI total score (an indicator for poor sleep quality) and higher scores on the *Hypomanic Core* subscale (rho = .158, *P* = 3E-11), and lower scores on the *Ordinariness* subscale (rho = -.165, *P* = 3E-12). Although correlations with *Social Vitality* were weaker (rho = -.063, *P* = .008), an exploratory linear regression analysis with ranked variables (resembling Spearman correlations) revealed that all three subscales explained independent variance in PSQI total scores. In total, 6.0% of the variance in PSQI total scores (adjusted for sex and age) was explained by the weighted combination of *Hypomanic Core* (β = .161, *P* = 1.3E-10, η_p_² = 0.023), *Social Vitality* (β = -.139, *P* = 8.6E-9, η_p_² = 0.019), and *Ordinariness* (β = -.135, *P* = 1.7E-8, η_p_² = 0.018). The strength of this association was substantial when compared to using the HPS total score (*HPS Total*) as the regressor, which resulted in an explained variance of only 0.4% (η_p_² = 0.004, *P* = .008). The observed failure of the *HPS Total* appeared to be attributable to the facet *Social Vitality.* Whereas the facet *Hypomanic Core* positively correlated with both *HPS Total* (rho = .832) and poor sleep quality and *Ordinariness* negatively correlated with both the *HPS Total* (rho = -.292) and poor sleep quality, *Social Vitality* correlated positively with *HPS Total* (rho = .721) but negatively with poor sleep quality. Hence, the multidimensional HPS scale differentially associates with subjective sleep quality, with associations of aggregated scales apparently cancelling each other out. To unravel the mechanisms between hypomanic personality and sleep, the differentiation of these facets is recommended.

**Prediction of HPS by sleep and covariates**

To investigate the relative importance of objective and subjective sleep variables and covariates in predicting Hypomanic Personality Scores, we carried out a series of regression analyses. In order to avoid overfitting and inflated R^2^ estimates, we used a tenfold cross-validation approach which involves evaluating the performance of a prediction model by applying the model to new data not used in training it.

We selected N = 640 subjects with valid data from both the actigraphy assessment and the PSQI questionnaire. From the set of 21 sleep variables, we dropped the actigraphy variable ‘mean sleep-offset time’ and the PSQI variable ‘sleep-efficiency’ due to reasons of multicollinearity (stepwise exclusion of variables with a variance inflation factor > 10).

We sought to determine the incremental predictive value of sleep variables by comparing the performance of a full model to the performance of a standard covariate model. The standard covariate model contained the predictors sex, age, socioeconomic status (SES), body mass index (BMI), and questionnaire-derived indicators of sleep apnea and Periodic Limb Movement Disorder (PLMD). We selected these variables due to their extensive implications for sleep behavior and affective traits. Socioeconomic status was calculated as a combination of education, working status, and household income as previously described.^15^ Information on sleep apnea and PLMD were drawn from two PSQI items that were answered by the participant’s partner and were not aggregated to the PSQI total score (‘long pauses between breaths while asleep’, ‘legs twitching or jerking while asleep’). These two PSQI items were rated on a 4-point scale. Missing item responses (22% and 20%, respectively) were substituted by the sample median.

We analyzed the performance of three different full models. The first full model contained the actigraphy variables, while the second model contained the PSQI variables. The third full model included both the actigraphy and PSQI variables. All full models included the variables of the standard covariate model as described above.

In order to carry out the tenfold cross-validation procedure, our sample was split into ten equal sized subsets. To ensure comparable distributions of HPS scores across the ten subsets, the sample was sorted according to the respective HPS score (total or subscale score) and subjects of the first, second, third, …, 64th decuplet were each randomly assigned to one of the ten subsets. Next, one of the subsets was selected as the testing dataset, while the other nine subsets served as the training dataset. After the first model was trained and tested, the next subset was selected as the testing sample, while the other nine subsets served as the training sample. This procedure was carried on until each subset served exactly once as the testing dataset. The tenfold cross-validation procedure with random group assignment was repeated 100 times so that 100 predictions were made for each subject. Predicted HPS scores were then averaged and correlated with the measured HPS scores.

Prediction models were built using the gradient boosting package ‘xgboost’ (v.0.82.1)^16^ in R. The xgboost algorithms have been widely used in the field of supervised machine learning and their solutions have been awarded in several data science competitions. We trained our models with the tree booster. The learning rate was set to eta = 0.02 with a maximum tree depth of 3. Training iterations were set to 1000. Of the nine training subsets, eight subsets served to fit the model, while one subset was selected to monitor model performance and avoid overfitting. The training process was stopped after 50 iterations if no further improvement was achieved. We used default settings for all other training parameters.

Supplementary Table S6 shows the Pearson correlations between predicted and measured HPS scores for the standard covariate model and the three full models. ΔR^2^ reflects the increase in explained variance of a full model relative to the standard covariate model and was calculated as follows:

ΔR^2^ = rho^2^_Full_ - rho^2^_Standard_

Significance of ΔR^2^ was calculated by comparing the correlations of the full and standard model using the formula by Dunn & Clark^12^ as implemented in the R package cocor v.1.1-3^13^.

**Supplementary Table S6**. Pearson correlations between predicted and measured HPS scores

|  | HPS total  sum-score | | | |  | HPS subscale Hypomanic Core | | | |  | HPS subscale Social Vitality | | | |  | HPS subscale Ordinariness | | | |
| --- | --- | --- | --- | --- | --- | --- | --- | --- | --- | --- | --- | --- | --- | --- | --- | --- | --- | --- | --- |
|  | rho | *p* | ΔR^2^ |  |  | rho | *p* | ΔR^2^ |  |  | rho | *p* | ΔR^2^ |  |  | rho | *p* | ΔR^2^ |  |
| Standard model | .015 | .696 |  |  |  | -.002 | .954 |  |  |  | .166 | 2E-5 |  |  |  | -.034 | .388 |  |  |
| Full model acti | .111 | .005 | 0.012 | * |  | .145 | 2E-4 | 0.021 | * |  | .162 | 4E-5 | -0.001 |  |  | .087 | .028 | 0.006 | * |
| Full model PSQI | .075 | .057 | 0.005 |  |  | .179 | 5E-6 | 0.032 | ** |  | .141 | 3E-4 | -0.008 |  |  | .066 | .095 | 0.003 | * |
| Full model acti + PSQI | .135 | .001 | 0.018 | * |  | .246 | 3E-10 | 0.060 | ** |  | .157 | 7E-5 | -0.003 |  |  | .113 | .004 | 0.012 | * |
| Predicted HPS scores were derived from a tenfold cross-validation procedure with 100 repeats. Models were trained using R package xgboost with booster type ‘gbtree’. ΔR^2^ reflects the increase in R^2^ relative to the standard model. Significance of ΔR^2^ is indicated by asterisks and was calculated by comparing the correlations of the full and standard model using the formula by Dunn & Clark^12^ as implemented in R package cocor v.1.1-3^13^.  * *p*(ΔR^2^) < .050 (two-sided nominal significance of ΔR^2^)  ** p(ΔR^2^) < .001 (two-sided nominal significance of ΔR^2^) | | | | | | | | | | | | | | | | | | | |
|  | | | | | | | | | | | | | | | | | | | |

Overall, adding objective and subjective sleep variables as features to the machine learning model significantly increased accuracy in predicting HPS scores. The highest predictive accuracy was achieved for the HPS subscale *Hypomanic Core*, resulting in a Pearson correlation between predicted and measured scores of rho = .246 (*p* = 3E-10) and an incremental value of sleep variables of ΔR^2^ = 0.060 (*p* = 7E-7). Furthermore, there was an incremental value of sleep variables in predicting both the subscale *Ordinariness* (ΔR^2^ = 0.012, *p* = .003) and the HPS total score (ΔR^2^ = 0.018, *p* = .010). In comparison, although predicted scores of the HPS subscale *Social Vitality* significantly correlated with the measured scores (rho = .157, *p* = 7E-5), analyses did not reveal evidence for an incremental value of sleep variables (ΔR^2^ = -0.003, *p* = .784). In sum, our analyses revealed a predictive value of sleep variables for three of the four HPS scores, with the highest model performance observed for the subscale *Hypomanic Core*.

Next, we extracted the relative contribution of each variable to the prediction models. We used the function ‘xgb.importance’ in the R package xgboost and averaged the relative feature gain across all full models that included both the actigraphy and PSQI variables. Supplementary Figure S5 shows the relative contribution of each variable to the respective prediction model.

**Supplementary Fig. S5.** Bar plot showing the relative importance of each variable to the prediction model


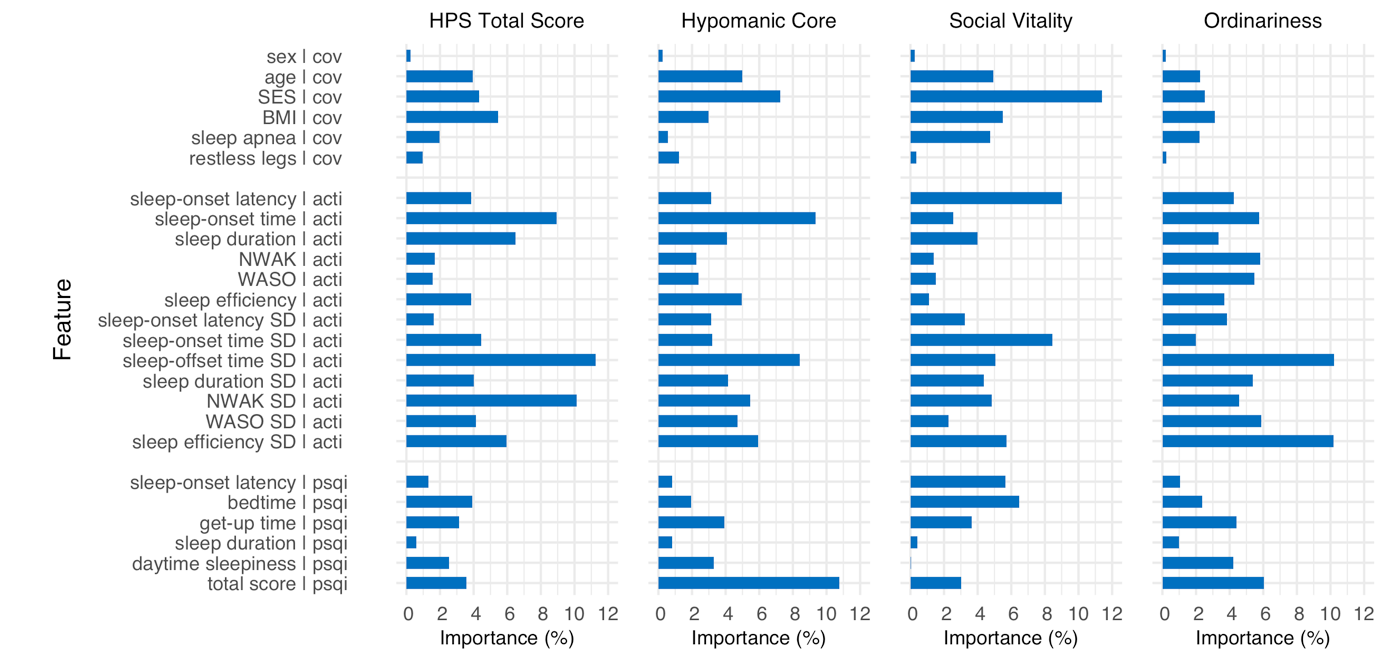


The bar plot illustrates the relative contribution of each feature to the tree-based prediction models. Note that the importance metric allows comparing the role of variables within each model, but does not enable inferences on absolute magnitudes across models. This implies that the bar plot does not account for varying prediction performances. BMI: Body Mass Index; SES: socioeconomic status; cov: covariate; acti: sleep variable from actigraphy; psqi: sleep variable from questionnaire Pittsburgh Sleep Quality Index; NWAK: number of awakenings; WASO: wake after sleep-onset time.

**Supplementary References**

1. Meyer, T. D., Drüke B. & Hautzinger M. Hypomane Persönlichkeit-Psychometrische Evaluation und erste Ergebnisse zur Validität der deutschen Version der Chapman-Skala. *Zeitschrift für Klinische Psychologie und Psychotherapie* **29**, 35-42 (2000).

2. Eckblad, M. & Chapman L. J. Development and validation of a scale for hypomanic personality. *J Abnorm Psychol* **95**, 214-222 (1986).

3. Revelle, W. psych: Procedures for Personality and Psychological Research. Northwestern University, Evanston, Illinois, USA. (2017).

4. Rawlings, D., Barrantes-Vidal N., Claridge G., McCreery C. & Galanos G. A factor analytic study of the Hypomanic Personality Scale in British, Spanish and Australian samples. *Personality and Individual Differences* **28**, 73-84 (2000).

5. Schalet, B. D., Durbin C. E. & Revelle W. Multidimensional structure of the Hypomanic Personality Scale. *Psychol Assess* **23**, 504-522 (2011).

6. Terrien, S., Stefaniak N., Morvan Y. & Besche-Richard C. Factor structure of the French version of the Hypomanic Personality Scale (HPS) in non-clinical young adults. *Compr Psychiatry* **62**, 105-113 (2015).

7. Geoffroy, P. A. et al. Sleep in patients with remitted bipolar disorders: a meta-analysis of actigraphy studies. *Acta Psychiatr Scand* **131**, 89-99 (2015).

8. Ng, T. H. et al. Sleep-wake disturbance in interepisode bipolar disorder and high-risk individuals: a systematic review and meta-analysis. *Sleep Med Rev* **20**, 46-58 (2015).

9. Morgan, J. F. p Value fetishism and use of the Bonferroni adjustment. *Evid Based Ment Health* **10**, 34-35 (2007).

10. Perneger, T. V. Adjusting for multiple testing in studies is less important than other concerns. *Bmj* **318**, 1288 (1999).

11. Benjamini, Y. & Hochberg Y. Controlling the False Discovery Rate: A Practical and Powerful Approach to Multiple Testing. *Journal of the Royal Statistical Society. Series B (Methodological)* **57**, 289-300 (1995).

12. Dunn, O. J. & Clark V. Correlation Coefficients Measured on the Same Individuals. *Journal of the American Statistical Association* **64**, 366-377 (1969).

13. Diedenhofen, B. & Musch J. cocor: a comprehensive solution for the statistical comparison of correlations. *PLoS ONE* **10**, e0121945 (2015).

14. Rosenthal, R. & DiMatteo M. R. Meta-analysis: recent developments in quantitative methods for literature reviews. *Annu Rev Psychol* **52**, 59-82 (2001).

15. Lampert, T., Kroll L. E., Müters S. & Stolzenberg H. Measurement of socioeconomic status in the German Health Interview and Examination Survey for Adults (DEGS1). *Bundesgesundheitsblatt* **56**, (2013).

16. Chen, T. et al. xgboost: Extreme Gradient Boosting. R package version 0.82.1. https://CRAN.R-project.org/package=xgboost (2019).
